# Chlorobenzene-driven palladium-catalysed lactonisation of benzoic acids†

**DOI:** 10.1039/d3ra08176a

**Published:** 2024-01-02

**Authors:** Masahiro Abe, Akiho Mizukami, Emi Yoshida, Tetsutaro Kimachi, Kiyofumi Inamoto

**Affiliations:** a School of Pharmacy and Pharmaceutical Sciences, Mukogawa Women's University 11-68, 9-Bancho, Koshien Nishinomiya Hyogo 663-8179 Japan abe_111@mukogawa-u.ac.jp inamoto@mukogawa-u.ac.jp

## Abstract

Herein, we developed a palladium-catalysed C–H cyclisation of benzoic acids in chlorobenzene without additional oxidants. The key to the success of these reactions is the use of chlorobenzene, which serves a dual role as a solvent and an oxidant, thus providing a simple and efficient method for synthesising phthalides.

## Abstract 2

Herein, we developed a palladium-catalysed C–H cyclisation of benzoic acids in chlorobenzene without additional oxidants.

## Graphical abstract

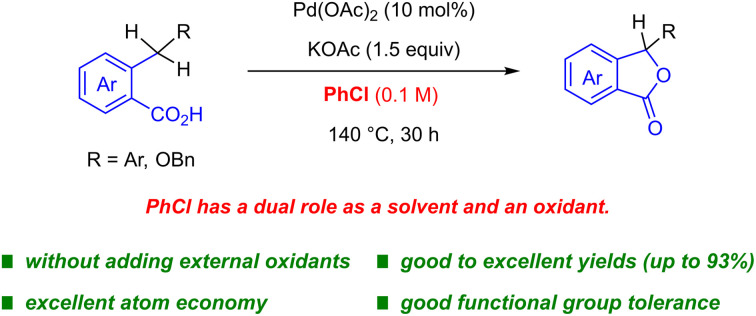

Chlorobenzene is a readily available and cost-effective feedstock and is thus commonly used as a solvent. Furthermore, chlorobenzene can enhance the oxidation of transition metals through oxidative addition^1^ and can be regarded as a suitable coupling partner.^2^ On the other hand, chlorobenzene itself has been rarely utilized as an external oxidant for transition-metal-catalysed C–H activation processes. In place of toxic metals and hazardous peroxides, the use of molecular oxygen or air as oxidants can be a viable option to achieve clean and economical conditions for C–H activations.^3^ Nevertheless, the substrate scope is often limited by oxygen-sensitive functionalities, such as a benzylic carbon centre, which might deteriorate through undesirable radical pathways. In addition, there is a risk associated with oxygen pressures when carrying out reactions in flammable organic solvents, especially in the case of a large-scale synthesis.^4^ Hence, employing chlorobenzene for transition-metal-catalysed C–H activations could offer not only a simplified protocol but also an alternative substrate scope compared with other chalcogenide oxidant-based C–H activations.

Phthalide scaffolds are found in many bioactive natural products and pharmaceutical agents, such as 3-butylphthalide,^5^ chrycolide,^6^ and noscapine (Fig. 1).^7^ Extensive research has been conducted on phthalide synthesis,^8,9^ with a focus on the lactonisation of benzoic acids through C–H activation, using various catalytic systems such as transition-metal catalysis,^10–13^ photocatalysis,^14^ electrolysis,^15^ and metal-free conditions (Scheme 1).^16^ For instance, Martin *et al.* reported a palladium-catalysed lactonisation of benzoic acids using stoichiometric silver salts as oxidants, yielding diversely substituted phthalides.^10^ Recently, Yu *et al.* achieved palladium-catalysed lactonisation using molecular oxygen as the sole oxidant under high pressure conditions.^11^ Despite the remarkable progress, most transition-metal catalysis-based methods required toxic and metallic oxidants in stoichiometric amounts, while other methods required high pressure conditions^11^ or specific reaction apparatus, such as photo- or electrochemical reactors.^14,15^ Herein, we describe a catalytic system driven by chlorobenzene involving palladium-catalysed C–H activation of benzoic acids under metallic oxidant-free conditions (Scheme 1). In this process, chlorobenzene serves a dual role as a solvent and an oxidant, resulting in the efficient and straightforward synthesis of variously substituted phthalides. Notably, our protocol enables the use of substrates sensitive to oxidation conditions, which should be an appealing feature of the method.

**Fig. 1:**
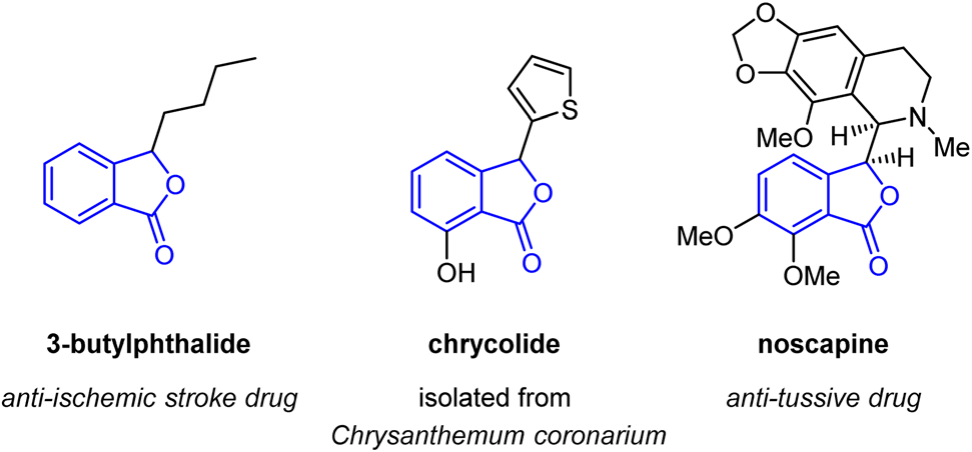
Phthalide scaffolds in natural products and pharmaceuticals.

**Scheme 1:**
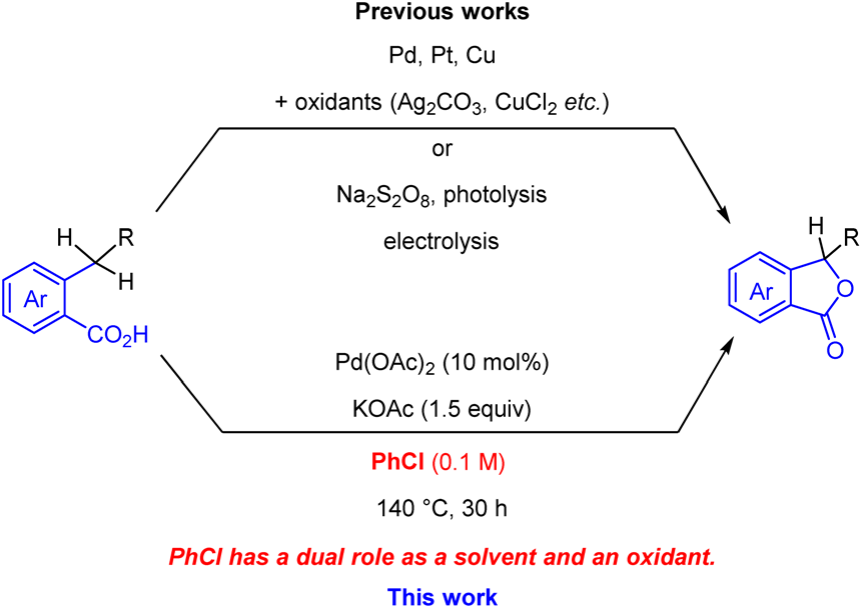
Palladium-catalysed phthalide syntheses.

Our study examined the cyclisation of 2-benzylbenzoic acid 1a using 10 mol% of Pd(OAc)_2_ with KOAc base as a model reaction (Table 1). As expected, chlorobenzene was the best solvent for the catalytic cycle, indicating its dual role as an oxidant and a solvent (entries 1 and 2). Subsequently, we evaluated various catalysts for the reaction (entries 3–5). While other palladium salts, such as PdCl_2_, Pd(TFA)_2_ and Pd(acac)_2_ yielded moderate yields (entry 3), several palladium complexes and other transition metals were ineffective (entries 4 and 5). Upon conducting a base screening, K_2_HPO_4_ demonstrated a similar result as KOAc, whereas other alkali metal bases led to lower yields (entries 6 and 7). The product yield was drastically improved by increasing the reaction temperature (entry 8). Moreover, the reaction was effectively scaled up by extending the reaction time (entries 9 and 10).

**Table 1:** Optimisation of reaction parameters^a^

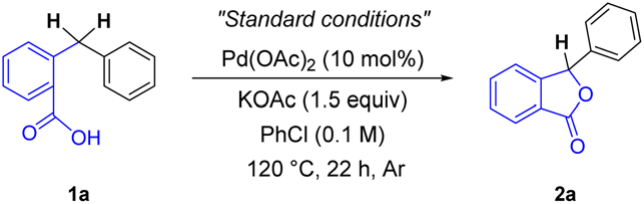		
Entry	Variation from “Standard conditions”	Yield^b^^,^^c^ (%)
1	None	59
2^d^	DMF, *p*-xylene, mesitylene instead of PhCl	0–36
3	PdCl_2_, Pd(TFA)_2_, Pd(acac)_2_ instead of Pd(OAc)_2_	21–51
4	PdCl_2_(tmeda), PdCl_2_(dppf), 10% Pd/C instead of Pd(OAc)_2_	0–5
5	Ni(OAc)_2_, NiCl_2_, CoCl_2_ instead of Pd(OAc)_2_	0
6	K_2_HPO_4_ instead of KOAc	59
7	LiOAc, NaOAc, Na_2_HPO_4_ instead of KOAc	14–33
8	140 °C instead of 120 °C	(82)
9^e^	On a 0.5 mmol scale instead of 0.2 mmol scale	70
10^e^^,^^f^	30 h instead of 22 h	(81)

a Reactions were run on a 0.2 mmol scale.

b Yields were determined by ^1^H NMR using an internal standard.

c Isolated yields are in parentheses.

d Reactions were run at 150 °C.

e Reaction was run at 140 °C.

f On a scale of 0.5 mmol scale.

Further, we investigated the substrate scope of our developed method (Table 2). First, various aromatic substituents at the 3-position on the phthalide ring were examined (2b–h). The installation of the electron-donating groups, such as Me and OMe, gave excellent yields of the desired phthalides 2b and 2c. Substitution with halogen atoms (F and Cl) was well tolerated during the reactions (2d and 2e). Furthermore, the naphthalene moiety was suitable for our process (2f). Notably, the reaction containing a substrate bearing a thiophene ring yielded the desired product 2g, which is the core structure of chrycolide. However, the sterically hindered substrate 1h did not undergo the desired transformation and only recovered the starting material. Then, various substituents at the 5-position on the phthalide ring, such as Me, Cl and CF_3_ groups, were well compatible for the process (2i–k). Additionally, we observed that a non-dibenzylic substrate 1l yielded the desired product 2l in a lower yield, while an alkyl-substituted substrate 1m and a bulky substrate 1n containing a tertiary carbon centre produced unsuccessful results. Finally, the reaction with 1a proved that scaling up to a 3 mmol was acceptable.

**Table 2:** Substrate scope^a^^,^^b^

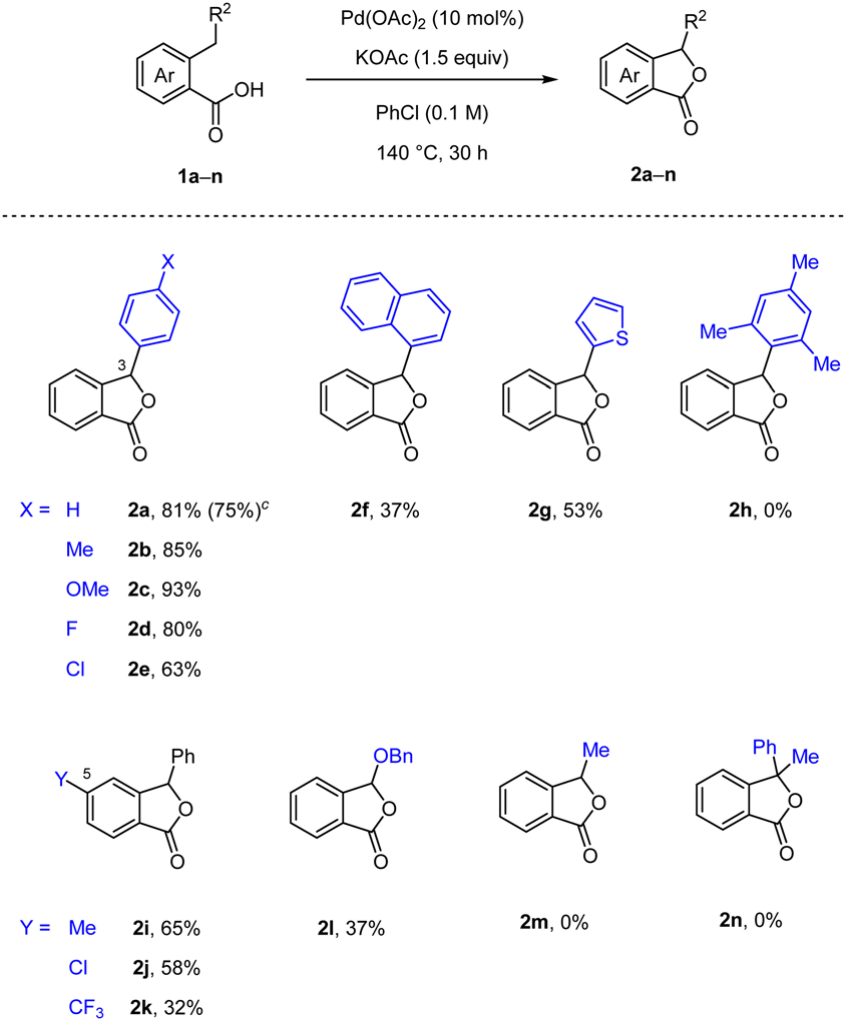

a Reactions were run on a 0.5 mmol scale.

b Isolated yields.

c Reaction was run on a 3.0 mmol scale in parentheses.

Preliminary experiments were conducted to investigate the reaction mechanism. The lactonisation occurred in the presence of 2,6-di-*t*-butyl-*p*-cresol (BHT) or galvinoxyl free radical as radical scavengers, indicating that radical pathways were not involved in the formation of 2a (Scheme 2). In addition, after the reaction of 1a, we detected a reasonable amount of benzene by GC analysis, which is likely produced from the protonation of a Pd–phenyl complex (Scheme 3). On the other hand, in the absent of Pd(OAc)_2_, the formation of benzene was not observed. Interestingly, when bromobenzene was used instead of chlorobenzene under optimised conditions, the desired product 2a with a 22% yield was obtained. The use of iodobenzene led to the formation of the *ortho*-arylated product 3 in 91% yield (Scheme 4).^17^ Furthermore, to showcase the synthetic utility of our process, we applied the Martin's conditions^10^ to substrate 1a. The desired product 2a was obtained only in 8% isolated yield, which was much lower than that obtained using our method (Scheme 5).

**Scheme 2:**
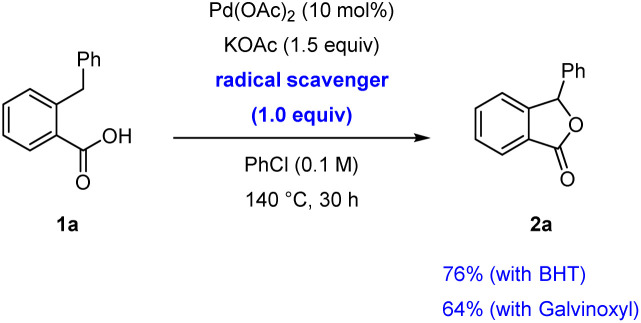
Reactions with radical scavengers.

**Scheme 3:**
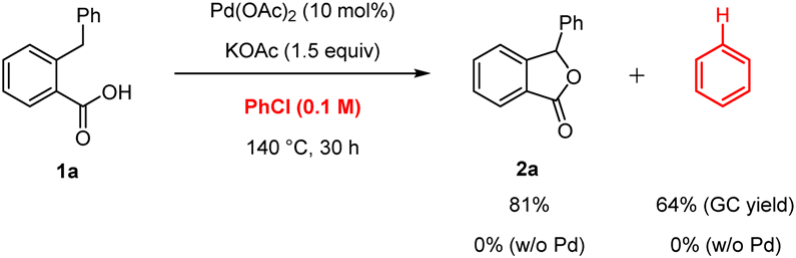
Detection of benzene in the reaction of 1a.

**Scheme 4:**
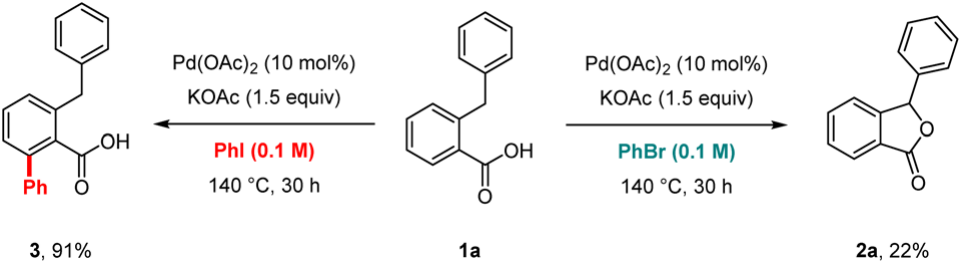
Reactions in halobenzenes.

**Scheme 5:**
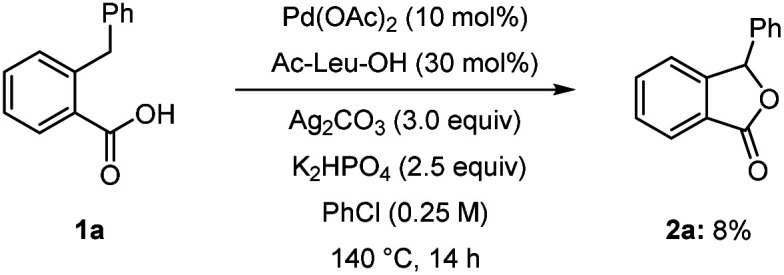
Martin's conditions for phthalide synthesis.

A plausible mechanism is proposed (Scheme 6). First, the potassium benzoate would coordinate with the palladium(ii) catalyst. Subsequently, intramolecular C–H activation *via* a concerted metalation-deprotonation pathway is presumed to occur. Further, two possible reaction pathways for the catalytic cycle are hypothesised. In one pathway, the palladacycle could undergo reductive elimination, yielding the desired phthalide and palladium (0). Another pathway, consistent with Musaev's report,^18^ is a stepwise S_N_2-type nucleophilic substitution pathway. The Pd–O bond cleavage of the palladacycle could generate a π–benzylic complex. Then, the nucleophilic attack of the carboxylate moiety on the benzylic carbon could provide the desired product and palladium (0). The palladium (0) would then undergo oxidative addition with chlorobenzene.

**Scheme 6:**
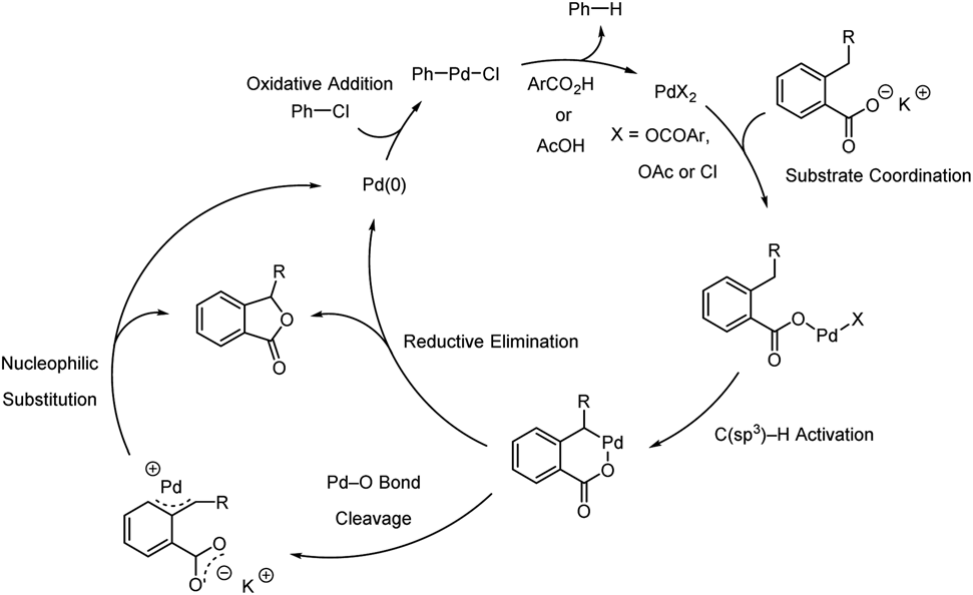
Plausible reaction mechanism.

In conclusion, we have successfully developed a chlorobenzene-driven C–H lactonisation in palladium catalysis for phthalide synthesis. Notably, our method eliminates the need for additional oxidants, providing a simple and easy-to-manipulate method for a biologically important phthalide nucleus. Our preliminary experiments on the reaction mechanism showed that the cyclization could not undergo radical pathways and chlorobenzene should be consumed for the oxidation of the palladium catalyst. Furthermore, we revealed that the use of chlorobenzene rather than bromo- and iodobenzene was crucial to the success of the reactions.

## Author contributions

M. A. and K. I. conceptualized and supervised the research and wrote the manuscript. A. M., E. Y. and T. K. conducted the investigation and prepared the ESI.†

## Conflicts of interest

There are no conflicts to declare.

## Supplementary Material

RA-014-D3RA08176A-s001
