# Supplementary material for: Chlorobenzene-driven palladium-catalysed lactonisation of benzoic acids
Source: RSC Adv. 2024 Jan 2;14(2):808–11. doi: 10.1039/d3ra08176a (PMC10759164; doi:10.1039/d3ra08176a)

Electronic Supplementary Material (ESI) for RSC Advances.
This journal is © The Royal Society of Chemistry 2023

Supplementary Information

Chlorobenzene-Driven Palladium-Catalysed Lactonisation of Benzoic Acids

Masahiro Abe,* Akiho Mizukami, Emi Yoshida, Tetsutaro Kimachi and Kiyofumi Inamoto*

School of Pharmacy and Pharmaceutical Sciences, Mukogawa Women's University 11-68, 9-Bancho, Koshien, Nishinomiya, Hyogo 663-8179, Japan

e-mail: abe_111@mukogawa-u.ac.jp, inamoto@mukogawa-u.ac.jp

1. General Comments **S2**

2. Materials **S2**

3. Details of Optimisation Studies **S3 – S7**

4. Negative Results for Phthalide Synthesis **S8**

5. Spectroscopic and Analytical Data **S9 – S15**

6. References **S15**

7. ^{1}H-, ^{13}C- and ^{19}F-NMR Spectra **S16 – S34**

1. General Comments

Melting points were measured with an AS ONE Corp. melting temperature measurement device (ATM-02) and uncorrected. IR spectra were recorded on a SHIMADZU IRAffinity-1. NMR data were recorded on either a JEOL JNM-ECP400 spectrometer (400 MHz) or a JEOL ECA500 spectrometer (500 MHz). Chemical shifts are expressed in δ (parts per million, ppm) values, and coupling constants are expressed in Hertz (Hz). ^{1}H NMR spectra were referenced to $(CH_3)_4Si$ (TMS) as an internal standard or to a residual proton signal in deuterated solvent ($CDCl_3$, 7.26 ppm). 1,1,2-Trichloroethane was used as an internal standard. ^{13}C NMR spectra were referenced to a residual proton signal in deuterated solvent ($CDCl_3$: 77.0 ppm). ^{19}F NMR spectra were referenced to 4-fluorotoluene as an internal standard (−118.0 ppm). The following abbreviations are used: s = singlet, d = doublet, t = triplet, q = quartet, dd, = double doublet, dt = double triplet, td = triple doublet, ddd = double double doublet, m = multiplet, and brs = broad signal. Mass spectra and high-resolution mass spectra were measured on a JEOL JMS-700 instrument. Chromatographic separations were achieved on silica gel columns (Wakosil C-200, 64 − 210 μm).

2. Materials

All commercially available materials including palladium(II) acetate (Fujifilm Wako Pure Chemical Corp., 169-07143), potassium acetate (Sigma-Aldrich Co., #791733), and chlorobenzene (Fujifilm Wako Pure Chemical Corp., 032-07986) were purchased from Sigma-Aldrich Co., Tokyo Chemical Industry Co., and Fujifilm Wako Pure Chemical Corp. and used as received. Test tubes with screw caps (IWAKI, TST SCR 18-180 and IWAKI, TST SCR 25-150) were used for the palladium-catalysed lactonisation. 2-benzylbenzoic acid **1a** and 2-ethylbenzoic acid **1m** were purchased from Sigma-Aldrich Co. (P36657) and Tokyo Chemical Industry Co. (E1347), and used as received. Starting materials **1b**,[1] **1c**,[2] **1d**,[3] **1e**,[4] **1f**,[5] **1g**,[3] **1l**[6] and **1n**[7] were prepared according to the literatures.

3. Details of Optimisation Studies

A. Screening of Palladium Catalysts

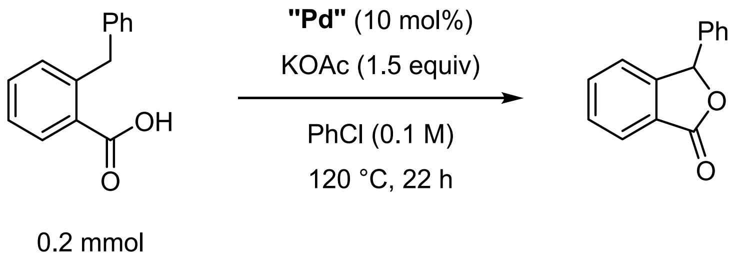

entry	"Pd"	yield (%)[a]
**1**	**$Pd(OAc)_2$**	**59**
2	$PdCl_2(PhCN)_2$	51
3	$Pd(acac)_2$	50
4	$Pd(TFA)_2$	21
5	$PdCl_2$	32
6	$PdCl_2(PPh_3)_2$	23
7	$PdCl_2$(dppp)	19
8	$PdCl_2$(tmeda)	0
9	$PdCl_2$(dppf)	0
10	(IPr)Pd(allyl)Cl	0
11	10% Pd/C	5
12	Pd_2dba_3 (5 mol%)	trace
13	none	0

[a]NMR yields.

B. Other Transition Metals

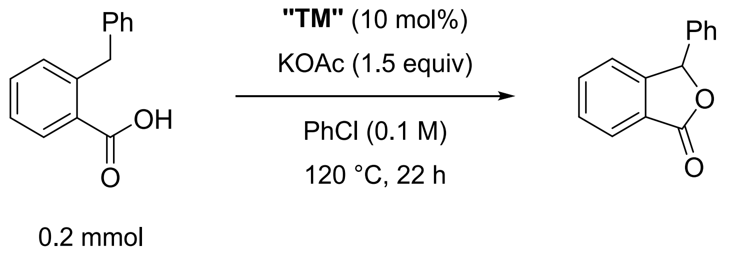

entry	"TM"	yield (%)[a]
1	$Ni(OAc)_2$	0
2	$NiCl_2$	0
3	$FeCl_3$	0
4	$CoCl_2$	0

[a]NMR yields.

C. Screening of Bases

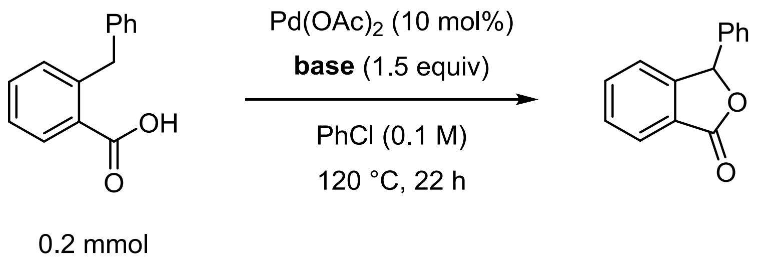

entry	base	yield (%)[a]
**1**	**KOAc**	**59**
2	NaOAc	33
3	LiOAc	14
4[b]	CsOAc	16
5	K_2HPO_4	59
6	Na_2HPO_4	8
7	NEt_3	0
8	pyridine	0

[a]NMR yields.
[b]Reaction was conducted at 140 °C for 30 h on a 0.5 mmol scale.

D. Amount of KOAc

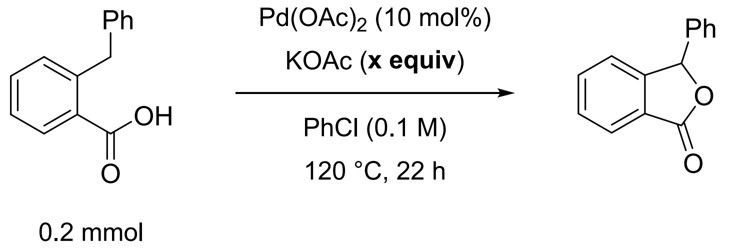

entry	x	yield (%)[a]
1	0.5	39
2	1.0	39
**3**	**1.5**	**59**
4	3.0	31
5	5.0	28
6	10	20

[a]NMR yields.

E. Solvent Effect (at 150 °C)

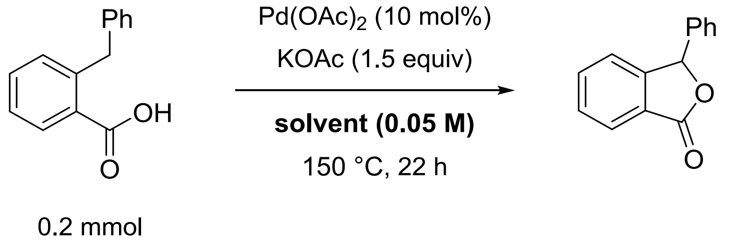

entry	solvent	yield (%)[a]
**1**	**PhCl**	**77**
2	*p*-xylene	36
3	*o*-xylene	22
4	*m*-xylene	25
5	mesitylene	14
6	DMA	0
7	DMSO	0
8	DMI	0

[a]NMR yields.

F. Reaction Temperature

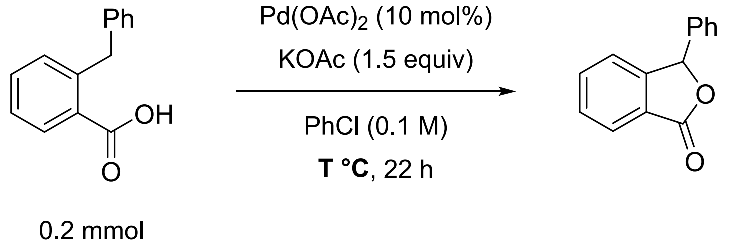

entry	T (°C)	yield (%)[a,b]
1	120	59
**2**	**140**	**90 (82)**
3	150	77

[a]NMR yields. [b]Isolated yield in parentheses.

G. Concentration (0.5 mmol Scale)

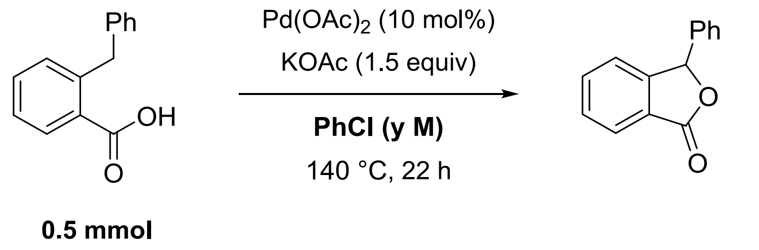

entry	y	yield (%)[a,b]
1	0.05	(59)
**2**	**0.1**	**70**
3	0.25	12

[a]NMR yields. [b]Isolated yield in parentheses.

H. Reaction Time

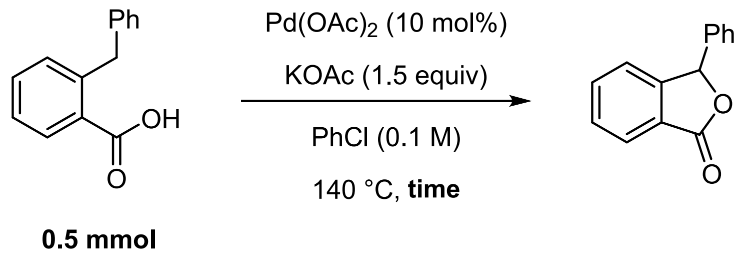

entry	time (h)	yield (%)[a,b]
1	22	70
**2**	**30**	**(81)**
3	48	(74)

[a]NMR yield. [b]Isolated yields in parentheses.

I. Amount of $Pd(OAc)_2$ and Reaction Time

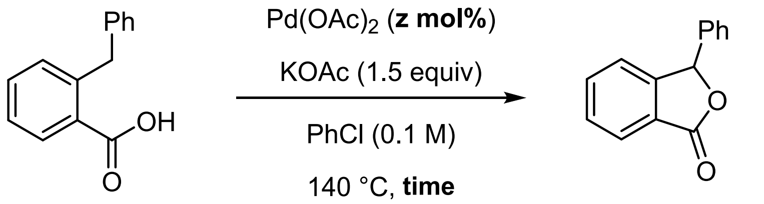

0.5 mmol

entry	z (mol%)	time (h)	yield (%)[a,b]
1	5	30	42
2	5	48	63
**3**	**10**	**30**	**(81)**

[a]NMR yield. [b]Isolated yields in parentheses.

J. Additive Effect

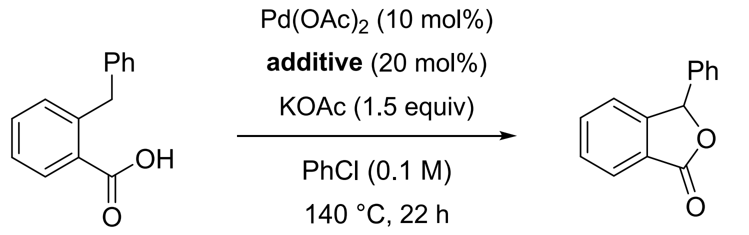

0.5 mmol

entry	additive	yield (%)[a]
**1**	**none**	**70**
2	cod	0
3	coe	25
4	cyclohexene	55
5	TBACl	9

[a]NMR yields.

K. In the Presence of Ag_2CO_3

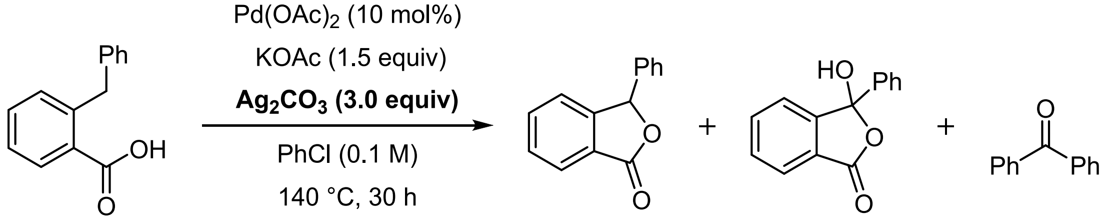

1a **2a:** 12% **2a':** 0% 0%

4. Negative Results for Phthalide Synthesis[a,b]

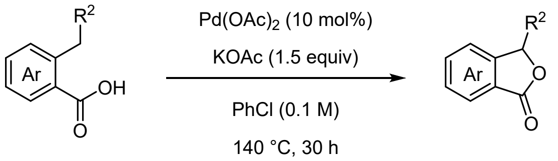

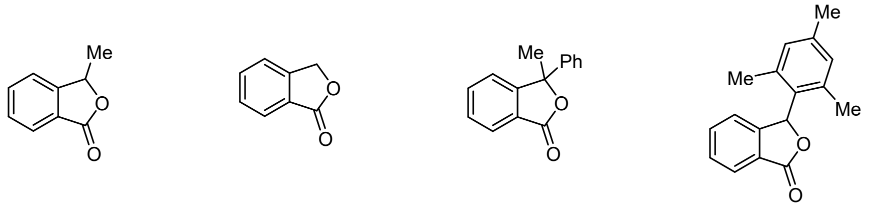

0% 0% 0% 0%

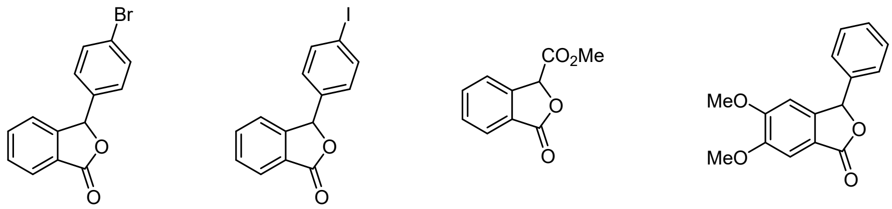

0% 0% 0% 0%

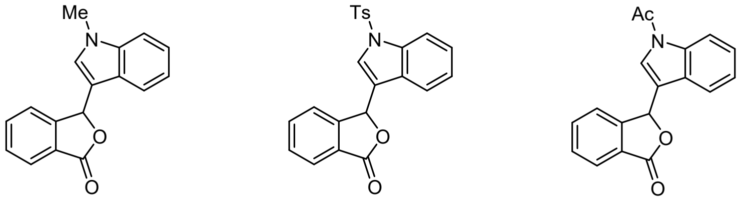

0% 0% 0%

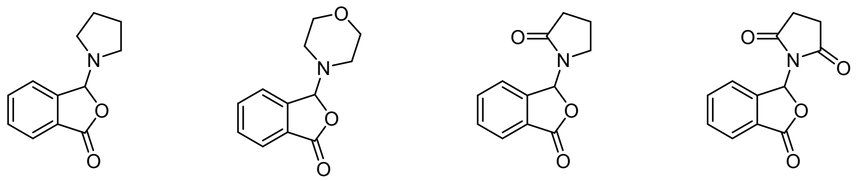

0% 0% 7% 0%

16%[c]

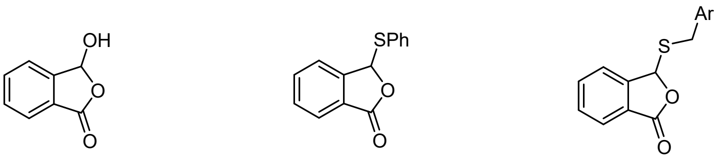

0% 0% 3% (Ar = *p*-*t*BuC_6H_4)

[a]Reactions were conducted on a 0.5 mmol scale.[b]Isolated yield. [c]CsOAc was used instead of KOAc.

5. Spectroscopic and Analytical Data

Typical procedure for phthalide synthesis

In a test tube, 2-benzylbenzoic acid **1a** (106.2 mg, 0.50 mmol), $Pd(OAc)_2$ (11.2 mg, 0.05 mmol, 10 mol%), and KOAc (73.6 mg, 0.75 mmol, 1.5 equiv) were added. The tube was evacuated and backfilled with Ar three times and then chlorobenzene (5 mL) was added. The tube was sealed and heated at 140 °C in an oil bath for 30 h. After cooling to room temperature, the reaction mixture was filtered through a short pad of silica gel and the filtrate was concentrated in vacuo. The crude was purified by silica gel column chromatography eluting with hexane/EtOAc (4:1) to afford 3-phenyl-3*H*-isobenzofuran-1-one (82% yield, 37.0 mg, 0.176 mmol) as a colorless solid.

3-Phenyl-3*H*-isobenzofuran-1-one (2a)[8]

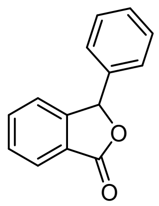

Yield 81% (85.2 mg, 0.405 mmol) from 2-benzylbenzoic acid **1a** (106.2 mg, 0.50 mmol); Eluent: hexane/EtOAc = 6:1; Colorless solid; ^{1}H NMR (400 MHz, $CDCl_3$/TMS) δ 7.96 (d, *J* = 7.5 Hz, 1H), 7.65 (td, *J* = 7.5, 1.2 Hz, 1H), 7.55 (t, *J* = 7.5 Hz, 1H), 7.42 – 7.31 (m, 4H), 7.30 – 7.25 (m, 2H), 6.40 (s, 1H); $^{13}C\{^1H\}$ NMR (101 MHz, $CDCl_3$/TMS) δ 170.6, 149.8, 136.5, 134.4, 129.5, 129.4, 129.1, 127.1, 125.74, 125.68, 123.0, 82.8; LRMS (EI) m/z: 210 $[M]^+$.

3-*p*-Tolyl-3*H*-isobenzofuran-1-one (2b)[8]

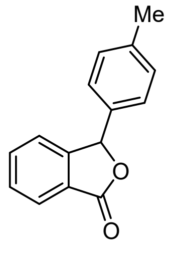

Yield 85% (95.4 mg, 0.425 mmol) from 2-(4-methylbenzyl)benzoic acid **1b** (113.0 mg, 0.50 mmol); Eluent: hexane/EtOAc = 6:1; Pale yellow solid; ^{1}H NMR (400 MHz, $CDCl_3$/TMS) δ 7.94 (d, *J* = 7.2 Hz, 1H), 7.63 (t, *J* = 7.2 Hz, 1H), 7.53 (t, *J* = 7.2 Hz, 1H), 7.31 (d, *J* = 7.2 Hz, 1H), 7.18 – 7.13 (m, 4H), 6.36 (s, 1H), 2.34 (s, 3H); $^{13}C\{^1H\}$ NMR (101 MHz, $CDCl_3$/TMS) δ 170.4, 149.8, 139.3, 134.2, 133.4, 129.6, 129.2, 127.0, 125.7, 125.5, 122.8, 82.7, 21.1; LRMS (EI) m/z: 224 $[M]^+$.

3-(4-Methoxyphenyl)-3*H*-isobenzofuran-1-one (2c)[8]

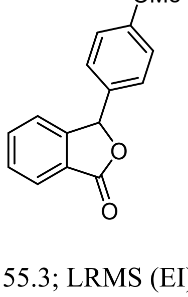

Yield 93% (112.3 mg, 0.467 mmol) from 2-(4-methoxybenzyl)benzoic acid **1c** (121.2 mg, 0.50 mmol); Eluent: hexane/EtOAc = 4:1; Colorless solid; ^{1}H NMR (400 MHz, $CDCl_3$/TMS) δ 7.96 (d, *J* = 7.2 Hz, 1H), 7.65 (t, *J* = 7.2 Hz, 1H), 7.56 (t, *J* = 7.2 Hz, 1H), 7.32 (d, *J* = 7.2 Hz, 1H), 7.18 (d, *J* = 8.8 Hz, 2H), 6.89 (d, *J* = 8.8 Hz, 2H), 6.37 (s, 1H), 3.80 (s, 3H); $^{13}C\{^1H\}$ NMR (101 MHz, $CDCl_3$/TMS) δ 170.5, 160.4, 149.7, 134.2, 129.3, 128.8, 128.3, 125.9, 125.5, 122.9, 114.3, 82.7, 55.3; LRMS (EI) m/z: 240 $[M]^+$.

3-(4-Fluorophenyl)-3*H*-isobenzofuran-1-one (2d)[8]

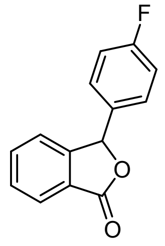

Yield 80% (90.5 mg, 0.400 mmol) from 2-(4-fluorobenzyl)benzoic acid **1d** (115.0 mg, 0.50 mmol); Eluent: hexane/EtOAc = 4:1; Pale yellow solid; ^{1}H NMR (400 MHz, $CDCl_3$/TMS) δ 7.95 (d, J = 7.2 Hz, 1H), 7.66 (t, J = 7.2 Hz, 1H), 7.56 (t, J = 7.2 Hz, 1H), 7.32 (d, J = 7.2 Hz, 1H), 7.27 – 7.23 (m, 2H), 7.08 – 7.03 (m, 2H), 6.39 (s, 1H); ^{13}C{^{1}H} NMR (101 MHz, $CDCl_3$/TMS) δ 170.1, 163.2 (d, $J_{C\text{-}F}$ = 247.8 Hz), 149.3, 134.4, 132.33, 132.30, 129.5, 129.0 (d, $J_{C\text{-}F}$ = 8.4 Hz), 125.6, 122.8, 115.9 (d, $J_{C\text{-}F}$ = 21.4 Hz), 81.9; ^{19}F NMR (376 MHz, $CDCl_3$) δ −111.1; LRMS (EI) m/z: 228 $[M]^+$.

3-(4-Chlorophenyl)-3*H*-isobenzofuran-1-one (2e)[8]

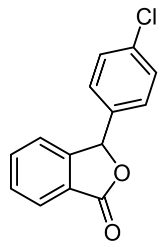

Yield 63% (76.9 mg, 0.314 mmol) from 2-(4-chlorobenzyl)benzoic acid **1e** (123.6 mg, 0.50 mmol); Eluent: hexane/EtOAc = 4:1; Colorless solid; ^{1}H NMR (400 MHz, $CDCl_3$/TMS) δ 7.95 (d, J = 7.6 Hz, 1H), 7.66 (t, J = 7.6 Hz, 1H), 7.56 (t, J = 7.6 Hz, 1H), 7.35 – 7.31 (m, 3H), 7.22 (d, J = 7.6 Hz, 2H), 6.37 (s, 1H); ^{13}C{^{1}H} NMR (101 MHz, $CDCl_3$/TMS) δ 170.1, 149.2, 135.2, 135.0, 134.4, 129.5, 129.1, 128.3, 125.7, 125.5, 122.7, 81.7; LRMS (EI) m/z: 244 $[M]^+$.

3-Naphthalen-1-yl-3*H*-isobenzofuran-1-one (2f)[8]

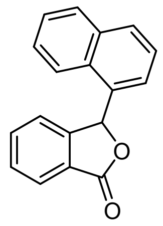

Yield 37% (48.3 mg, 0.186 mmol) from 2-naphthalen-1-ylmethylbenzoic acid **1f** (130.7 mg, 0.50 mmol); Eluent: hexane/EtOAc = 6:1; Brown solid; ^{1}H NMR (400 MHz, $CDCl_3$/TMS) δ 8.21 (d, J = 8.0 Hz, 1H), 7.99 (d, J = 7.6 Hz, 1H), 7.91 (d, J = 8.0 Hz, 1H), 7.85 (d, J = 7.6 Hz, 1H), 7.63 – 7.53 (m, 4H), 7.41 – 7.35 (m, 2H), 7.25 (d, J = 8.0 Hz, 1H), 7.21 (s, 1H); ^{13}C{^{1}H} NMR (101 MHz, $CDCl_3$/TMS) δ 170.4, 149.3, 134.1, 134.0, 131.9, 131.3, 129.9, 129.4, 129.0, 127.0, 126.2, 126.1, 125.9, 125.2, 124.5, 123.1, 122.9, 79.6; LRMS (EI) m/z: 260 $[M]^+$.

3-Thiophen-2-yl-3*H*-isobenzofuran-1-one (2g)[3]

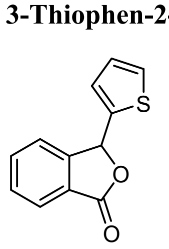

Yield 53% (56.8 mg, 0.263 mmol) from 2-thiophen-2-ylmethylbenzoic acid **1g** (109.2 mg, 0.50 mmol); Eluent: hexane/THF without BHT = 10:1; Colorless solid; ^{1}H NMR (400 MHz, $CDCl_3$/TMS) δ 7.93 (dt, J = 7.2, 1.2 Hz, 1H), 7.70 (td, J = 7.2, 1.2 Hz, 1H), 7.60 (tt, J = 7.2, 0.8 Hz, 1H), 7.49 – 7.46 (m, 1H), 7.37 (dd, J = 4.8, 1.2 Hz, 1H), 7.15 – 7.14 (m, 1H), 7.04 – 7.01 (m, 1H), 6.67 (s, 1H); ^{13}C{^{1}H} NMR (126 MHz, $CDCl_3$/TMS) δ 169.6, 148.6, 138.9, 134.2, 129.7, 127.8, 127.4, 127.0, 125.8, 125.6, 123.1, 77.8; LRMS (EI) m/z: 216 $[M]^+$.

5-Methyl-3-phenyl-3*H*-isobenzofuran-1-one (2i)

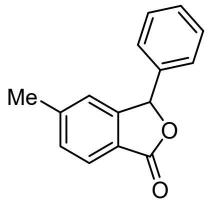

Yield 65% (79.7 mg, 0.326 mmol) from 2-benzyl-4-methylbenzoic acid **1i** (112.9 mg, 0.50 mmol); Eluent: hexane/EtOAc = 4:1; Colorless solid, m.p. 133 – 135 °C (hexane/EtOAc); ^{1}H NMR (400 MHz, $CDCl_3$/TMS) δ 7.83 (d, *J* = 8.0 Hz, 1H), 7.38 – 7.33 (m, 4H), 7.28 – 7.26 (m, 2H), 7.10 (s, 1H), 6.33 (s, 1H), 2.43 (s, 3H); $^{13}C\{^{1}H\}$ NMR (126 MHz, $CDCl_3$/TMS) δ 170.4, 150.3, 145.6, 136.7, 130.5, 129.2, 128.9 (2C), 126.9, 125.4, 123.1, 82.4, 22.0; IR (neat): 3063, 3034, 2959, 1746 cm^{-1}; LRMS (EI) m/z: 224 $[M]^+$; HRMS (EI-TOF) m/z: $[M]^+$ calcd for $C_{15}H_{12}O_2$ 224.0837; found 224.0839.

5-Chloro-3-phenyl-3*H*-isobenzofuran-1-one (2j)

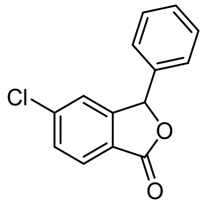

Yield 58% (71.0 mg, 0.290 mmol) from 2-benzyl-4-chlorobenzoic acid **1j** (123.0 mg, 0.50 mmol); Eluent: hexane/EtOAc = 4:1; Colorless solid, m.p. 184 – 185 °C (hexane/EtOAc); ^{1}H NMR (400 MHz, $CDCl_3$/TMS) δ 7.87 (d, *J* = 8.0 Hz, 1H), 7.51 (dd, *J* = 8.0, 1.2 Hz, 1H), 7.38 (m, 3H), 7.31 (s, 1H), 7.27 – 7.25 (m, 2H), 6.36 (s, 1H); $^{13}C\{^{1}H\}$ NMR (101 MHz, $CDCl_3$/TMS) δ 169.2, 151.3, 141.0, 135.7, 130.1, 129.5, 129.1, 126.8, 126.7, 124.1, 123.2, 82.0; IR (neat): 3084, 3069, 3032, 2970, 1746 cm^{-1}; LRMS (EI) m/z: 244 $[M]^+$; HRMS (EI-TOF) m/z: $[M]^+$ calcd for $C_{14}H_9{}^{35}ClO_2$ 244.0291; found 244.0289.

3-Phenyl-5-trifluoromethyl-3*H*-isobenzofuran-1-one (2k)

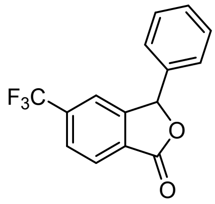

Yield 32% (44.2 mg, 0.159 mmol) from 2-benzyl-4-trifluoromethylbenzoic acid **1k** (140.6 mg, 0.50 mmol); Eluent: hexane/EtOAc = 4:1; Colorless solid, m.p. 112 – 114 °C (hexane/EtOAc); ^{1}H NMR (400 MHz, $CDCl_3$/TMS) δ 8.09 (d, *J* = 8.0 Hz, 1H), 7.83 (d, *J* = 8.0 Hz, 1H), 7.60 (s, 1H), 7.43 – 7.41 (m, 3H), 7.29 – 7.27 (m, 2H), 6.46 (s, 1H); $^{13}C\{^{1}H\}$ NMR (101 MHz, $CDCl_3$/TMS) δ 168.9, 150.1, 136.2 (q, $J_{C\text{–}F}$ = 32.9 Hz), 135.4, 129.7, 129.2, 128.9, 126.9, 126.7 (q, $J_{C\text{–}F}$ = 3.0 Hz), 126.4, 123.1 (q, $J_{C\text{–}F}$ = 272.2 Hz), 120.3 (q, $J_{C\text{–}F}$ = 3.9 Hz), 82.7; ^{19}F NMR (376 MHz, $CDCl_3$) δ −62.1; IR (neat): 3063, 3034, 2945, 1753 cm^{-1}; LRMS (EI) m/z: 278 $[M]^+$; HRMS (EI-TOF) m/z: $[M]^+$ calcd for $C_{15}H_9F_3O_2$ 278.0555; found 278.0552.

3-Benzyloxy-3*H*-isobenzofuran-1-one (2l)[9]

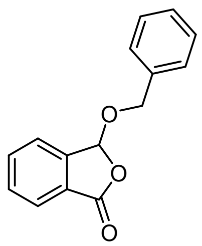

Yield 37% (44.0 mg, 0.183 mmol) from 2-benzyloxymethylbenzoic acid **1l** (121.5 mg, 0.50 mmol); Eluent: hexane/EtOAc = 6:1; Colorless solid; ^{1}H NMR (400 MHz, $CDCl_3$/TMS) δ 7.88 (d, *J* = 7.2 Hz, 1H), 7.68 (t, *J* = 7.2 Hz, 1H), 7.60 – 7.54 (m, 2H), 7.42 – 7.31 (m, 5H), 6.42 (s, 1H), 4.95 (d, *J* = 11.2 Hz, 1H), 4.83 (d, *J* = 11.2 Hz, 1H); $^{13}C\{^{1}H\}$ NMR (101 MHz, $CDCl_3$/TMS) δ 168.6, 145.0,

136.1, 134.3, 130.8, 128.6, 128.33, 128.28, 127.2, 125.4, 123.4, 101.1, 71.4; LRMS (EI) m/z: 240 $[M]^+$.

3-Benzylbiphenyl-2-carboxylic acid (3)

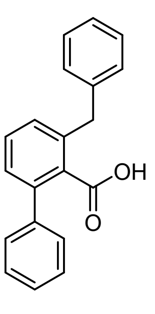

Yield 91% (130.5 mg, 0.453 mmol) from 2-benzylbenzoic acid **1a** (106.4 mg, 0.50 mmol); Eluent: hexane/EtOAc = 4:1; Colorless solid, m.p. 119 – 121 °C, (hexane/EtOAc); ^{1}H NMR (400 MHz, $CDCl_3$/TMS) δ 7.41 – 7.38 (m, 2H), 7.36 – 7.33 (m, 2H), 7.32 – 7.27 (m, 2H), 7.25 – 7.22 (m, 3H), 7.20 – 7.16 (m, 3H), 7.13 (dd, J = 7.6, 0.8 Hz, 1H), 4.11 (s, 2H); ^{13}C{^{1}H} NMR (101 MHz, $CDCl_3$/TMS) δ 174.6, 140.5, 140.4, 139.9, 138.6, 132.1, 129.8, 129.2, 129.0, 128.5, 128.4, 128.3, 128.0, 127.6, 126.3, 39.2; IR (neat): 3022, 2913, 2656, 2550, 1697 cm^{-1}; LRMS (EI) m/z: 288 $[M]^+$; HRMS (EI-TOF) m/z: $[M]^+$ calcd for $C_{20}H_{16}O_2$ 288.1150; found 288.1152.

Synthesis of 2-(2,4,6-Trimethylbenzyl)benzoic acid (1h)

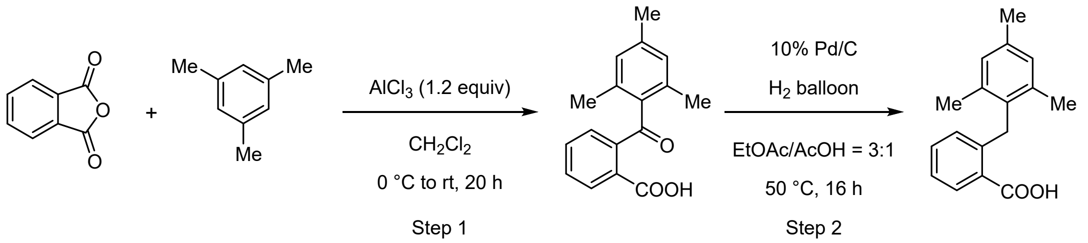

Step 1: To a solution of phthalic anhydride (5.0 g, 33.8 mmol 1.0 equiv) and mesitylene (5.6 mL, 40.5 mmol, 1.2 equiv) in CH_2Cl_2 (60 mL) at 0 °C was added anhydrous $AlCl_3$ (5.4 g, 40.5 mmol, 1.2 equiv) in 6 portions. The mixture was stirred at room temperature for 20 h. The mixture was cooled to 0 °C and quenched carefully with 1M HCl aq. (100 mL). The mixture was extracted with CH_2Cl_2 (100 mL × 3) and the combined organic layers were dried over $MgSO_4$, filtered, and concentrated under a reduced pressure. The crude was purified by recrystallization with hexane/EtOAc to afford the desired benzoic acid (54%, 4.90 g, 18.3 mmol).

Step 2: In a flask, the benzoic acid (1.07 g, 4.0 mmol, 1.0 equiv) and 10% Pd/C (0.50 g, 0.47 mmol, 0.12 equiv) were dissolved with EtOAc (30 mL) and AcOH (10 mL). The flask was evacuated and refilled with H_2 three times using a balloon. The mixture was stirred at 50 °C for 16 h. After cooling to room temperature, the mixture was filtered by Celite®, washed with EtOAc, and evaporated *in vacuo*. The crude was purified by silica gel column chromatography (hexane/EtOAc = 10:1 to 4:1) to afford the benzoic acid **1h** in 93% yield (943.4 mg, 3.71 mmol).

Colorless solid, m.p. 217 – 219 °C (hexane/EtOAc); ^{1}H NMR (400 MHz, $CDCl_3$/TMS) δ 8.13 (d, J =

6.8 Hz, 1H), 7.33 (t, *J* = 6.8 Hz, 1H), 7.26 (t, *J* = 7.6 Hz, 1H), 6.91 (s, 2H), 6.71 (d, *J* = 7.6 Hz,1H), 4.47 (s, 2H), 2.31 (s, 3H), 2.15 (s, 6H); $^{13}C\{^1H\}$ NMR (101 MHz, $CDCl_3$/TMS) δ173.2, 143.1, 137.3, 135.8, 133.3, 133.2, 131.7, 128.9, 128.3, 128.1, 125.8, 33.1, 20.9, 19.9; IR (neat) 3063, 2968, 2914, 2857, 2810, 2635, 1674 cm^{-1}; LRMS (EI) m/z: 254 $[M]^+$; HRMS (EI-TOF) m/z: $[M]^+$ calcd for $C_{17}H_{18}O_2$ 254.1307; found 254.1307.

General procedure for benzoic acid synthesis (1i–k)

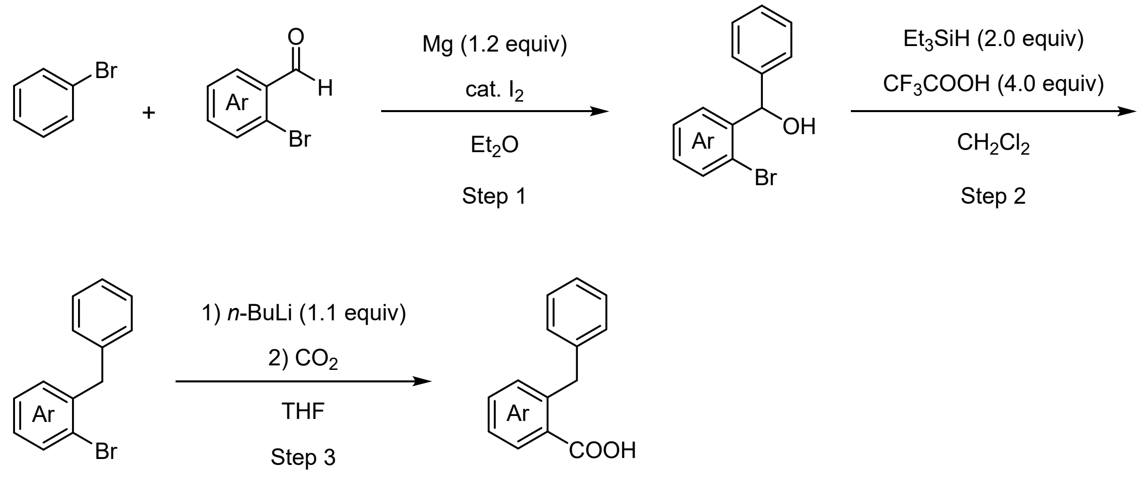

1i–k

Step 1: A solution of bromobenzene (1.5 equiv) in Et_2O (0.25 M) was added slowly to the mixture of Mg turnings (1.2 equiv) with a small amount of I_2 (0.01 equiv), and the mixture was stirred for 1 h. A solution of an aldehyde (1.0 equiv) in Et_2O (0.10 M) was added to the reaction mixture slowly at −20 °C. The resultant mixture was stirred at −20 °C until the reaction was completed as monitored by TLC (for 4–5 h). The reaction mixture was slowly diluted with sat. NH_4Cl aq. (10 mL), and extracted with EtOAc (20 mL × 3). The combined organic layers were dried over $MgSO_4$ and concentrated under a reduced pressure. The residue was purified by silica gel column chromatography to give the desired alcohol.

Step 2: To a solution of the alcohol (1.0 equiv) in CH_2Cl_2 (0.20 M) was added CF_3CO_2H (4.0 equiv) dropwise at 0 °C. After stirred for 10 min, Et_3SiH (2.0 equiv) was added dropwise, and the resulting mixture was stirred at rt overnight. The solvent was concentrated under a reduced pressure, and the residue was purified by silica gel column chromatography to give the desired aryl bromide.

Step 3: To a solution of an aryl bromide (1.0 equiv) in THF (0.20 M) was added *n*-BuLi (1.6 M, 1.1 equiv) dropwise at −78 °C. The reaction mixture was stirred at −78 °C for 1 h. Anhydrous CO_2 was bubbled through the mixture for 30 min. The reaction mixture was allowed to warm to rt for 30 min. (In case of the synthesis of **1i**): The reaction was quenched with H_2O (10 mL), basified with 1M NaOH

aq. to pH 12–14, and washed with EtOAc (10 mL). The resulting aqueous layer was acidified with HCl aq. to pH 1–2 and extracted with CH_2Cl_2 (20 mL × 2). The combined organic layers were washed with water (20 mL) and brine (20 mL), then dried over $MgSO_4$ and concentrated under a reduced pressure. The residue was recrystallized with hexane/EtOAc to give the desired carboxylic acid **1i.**

(In case of the synthesis of **1j**): The reaction was quenched with H_2O (10 mL), basified with 1M NaOH aq. to pH 12–14, and washed with EtOAc (10 mL). The resulting aqueous layer was acidified with HCl aq. to pH 1–2 and extracted with CH_2Cl_2 (20 mL × 2). The combined organic layers were washed with water (20 mL) and brine (20 mL), then dried over $MgSO_4$ and concentrated under a reduced pressure. The residue was purified by silica gel column chromatography to give the desired carboxylic acid **1j.**

(In case of the synthesis of **1k**): The reaction was quenched with H_2O (10 mL), basified with 1M NaOH aq. to pH 12–14, and extracted with EtOAc (10 mL × 1). The organic layer was dried over $MgSO_4$ and concentrated under a reduced pressure. The residue was purified by silica gel column chromatography to give the desired carboxylic acid **1k**.

2-Benzyl-4-methylbenzoic acid (1i)

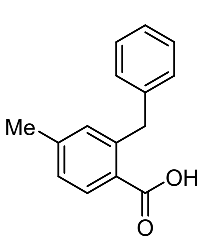

Yield 74% over 3 steps (756.0 mg, 3.34 mmol) from 2-bromo-5-methylbenzaldehyde (1.0 g, 5.02 mmol); Eluent: hexane/EtOAc = 50:1 to 4:1 (step 1) and hexane/EtOAc = 50:1 (step 2) and recrystallization with hexane/EtOAc (step 3); Colorless solid, m.p. 122 – 125 °C (hexane/EtOAc); ^1H NMR (400 MHz, $CDCl_3$/TMS) δ 7.96 (d, J = 8.0 Hz, 1H), 7.27 – 7.24 (m, 3H), 7.19 – 7.15 (m, 3H), 7.12 – 7.10 (m, 1H), 7.04 (s, 1H), 4.42 (s, 2H), 2.34 (s, 3H); $^{13}C\{^1H\}$ NMR (101 MHz, $CDCl_3$/TMS) δ 172.8, 143.7, 143.6, 140.9, 132.6, 132.0, 129.0, 128.3, 127.1, 125.9, 125.6, 39.6, 21.5; IR (neat): 2970, 2860, 2812, 2637, 1682 cm^{-1}; LRMS (EI) m/z: 226 $[M]^+$; HRMS (EI-TOF) m/z: $[M]^+$ calcd for $C_{15}H_{14}O_2$ 226.0994; found 226.0996.

2-Benzyl-4-chlorobenzoic acid (1j)

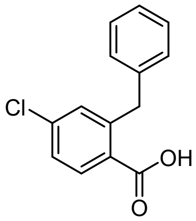

Yield 25% over 3 steps (570.2 mg, 2.31 mmol) from 2-bromo-5-chlorobenzaldehyde (2.0 g, 9.11 mmol); Eluent: hexane/EtOAc = 4:1 (step 1), hexane/EtOAc = 50:1 (step 2) and hexane/EtOAc = 2:1 (step 3); Colorless solid, m.p. 149 – 150 °C (hexane/ EtOAc); ^1H NMR (400 MHz, $CDCl_3$/TMS) δ7.99 (d, J = 8.4 Hz, 1H), 7.30 – 7.26 (m, 3H), 7.22 – 7.19 (m, 2H), 7.16 – 7.14 (m, 2H), 4.42 (s, 2H); $^{13}C\{^1H\}$ NMR (101 MHz, $CDCl_3$/TMS) δ 172.3, 145.7, 139.7, 139.5, 133.2, 131.7, 129.0 (2C), 128.5, 126.7, 126.3, 39.4; IR (neat): 3030, 2818, 2654, 2524, 1682 cm^{-1}; LRMS (EI) m/z: 246 $[M]^+$; HRMS (EI-TOF) m/z: $[M]^+$ calcd for $C_{14}H_{11}{}^{35}ClO_2$ 246.0448; found 246.0450.

2-Benzyl-4-trifluoromethylbenzoic acid (1k)

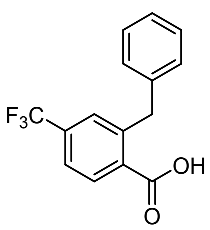

Yield 19% over 3 steps (209.0 mg, 0.745 mmol) from 2-bromo-5-trifluoromethylbenzaldehyde (1.0 g, 3.95 mmol); Eluent: hexane/EtOAc = 50:1 to 4:1 (step 1), hexane/EtOAc = 50:1 (step 2) and hexane/EtOAc = 4:1 (step 3); Colorless solid, m.p. 120 – 123 °C (hexane/EtOAc); ^{1}H NMR (400 MHz, $CDCl_3$/TMS) δ 8.14 (d, J = 8.0 Hz, 1H), 7.57 (d, J = 8.0 Hz, 1H), 7.50 (s, 1H), 7.30 – 7.24 (m, 2H), 7.22 – 7.18 (m, 1H), 7.14 – 7.12 (m, 2H), 4.48 (s, 2H); ^{13}C{^{1}H} NMR (101 MHz, $CDCl_3$/TMS) δ 171.9, 144.3, 139.5, 134.4 (q, $J_{C–F}$ = 32.1 Hz), 132.1, 131.8, 128.9, 128.6, 128.4 (q, $J_{C–F}$ = 3.8 Hz), 126.5, 123.4, 123.3 (q, $J_{C–F}$ = 272.2 Hz), 39.5; ^{19}F NMR (376 MHz, $CDCl_3$) δ −62.6; IR (neat): 3032, 2797, 2650, 2525, 1697 cm^{-1}; LRMS (EI) m/z: 280 $[M]^+$; HRMS (EI-TOF) m/z: $[M]^+$ calcd for $C_{15}H_{11}F_3O_2$ 280.0711; found 280.0708.

6. References

1. Y. Wang, S. Yang, G. Bian and L. Song, *Synthesis,* 2017, **49**, 1884.
2. A. K. Turek, D. J. Hardee, A. M. Ullman, D. G. Nocera and E. N. Jacobsen, *Angew. Chem. Int. Ed.,* 2016, **55**, 539.
3. T. Duhamel and K. Muniz, *Chem. Commun*., 2019, **55**, 933.
4. G. L. Regina, M. Puxeddu, M. Nalli, D. Vullo, P. Gratteri, C. T Supuran, A. Nocentini and R. Silvestri, *ACS Med. Chem. Lett*., 2020, **11**, 633.
5. M. Abe, K. Ueta, S. Tanaka, T. Kimachi and K. Inamoto, *RSC Adv*., 2021, **11**, 26988.
6. W. Kirmse and K. Kund, *J. Am. Chem. Soc.*, 1989, **111**, 1465.
7. K. Nozawa-Kumada, K. Ono, S. Kurosu, M. Shigeno and Y. Kondo, *Org. Biomol. Chem*., 2022, **20**, 5948.
8. Z. Ye, P. Qian, G. Lv, F. Luo and J. Cheng, *J. Org. Chem*., 2010, **75**, 6043.
9. T. Matsuda, K. Suzuki, S. Abe, H. Kirikae and N. Okada, *Tetrahedron*, 2015, **71**, 9264.

6. ^{1}H-, ^{13}C- and ^{19}F-NMR Spectra

^{1}H NMR (400 MHz, $CDCl_3$) of **2a**

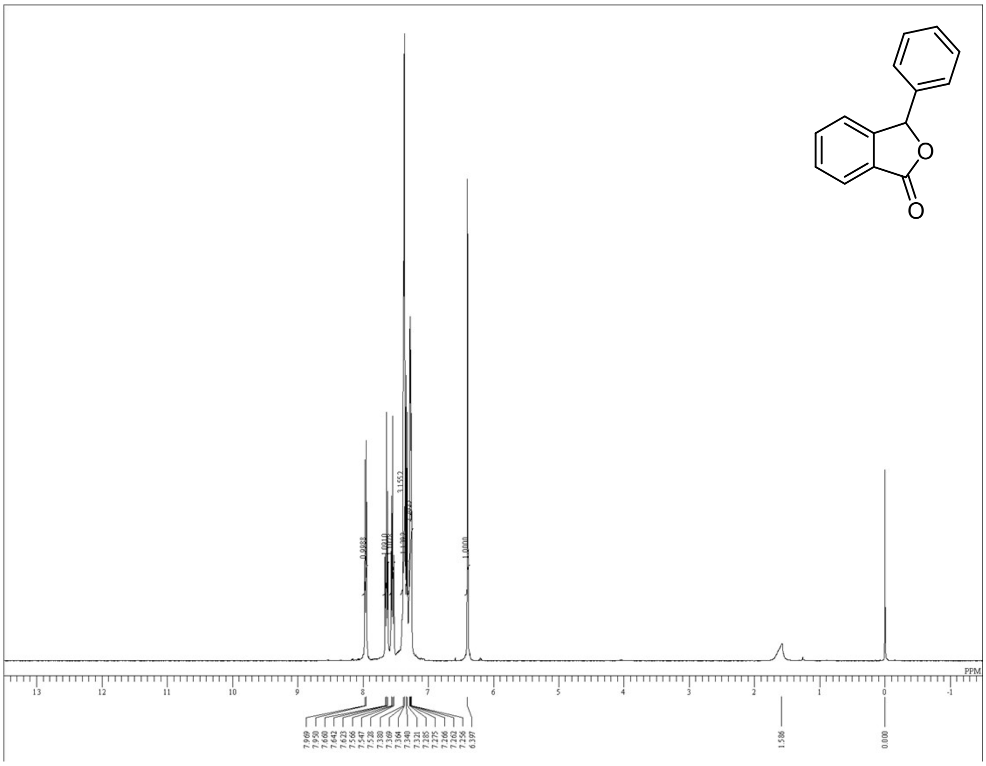

^{13}C{^{1}H} NMR (101 MHz, $CDCl_3$) of **2a**

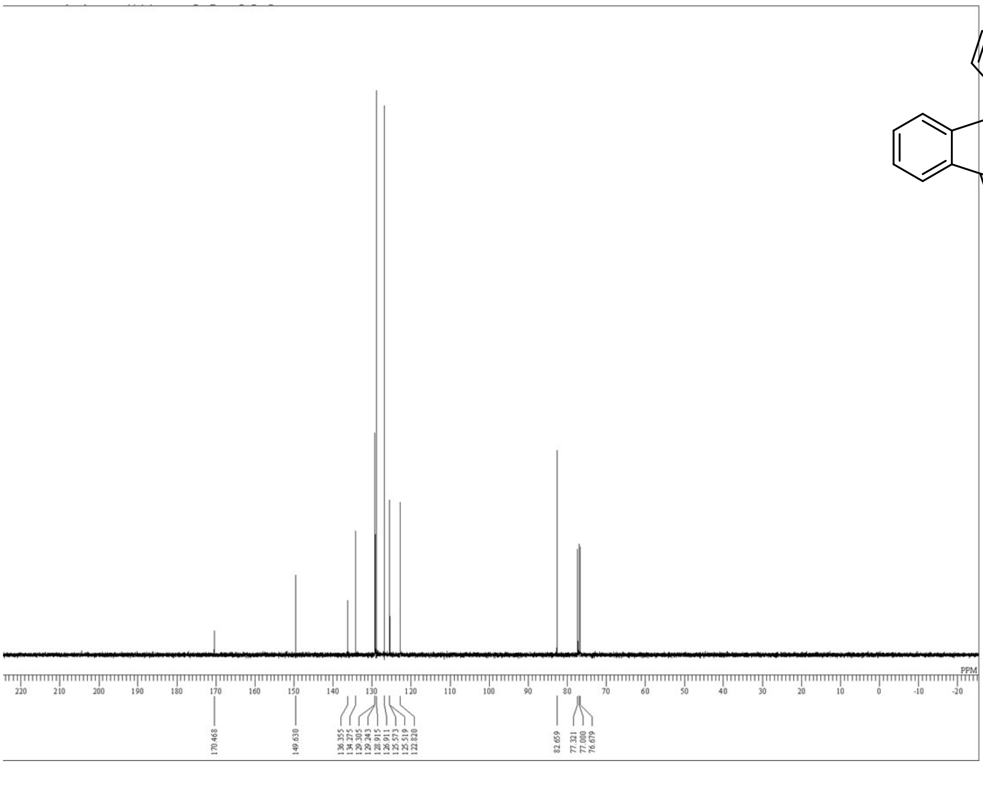

^{1}H NMR (400 MHz, $CDCl_3$) of **2b**

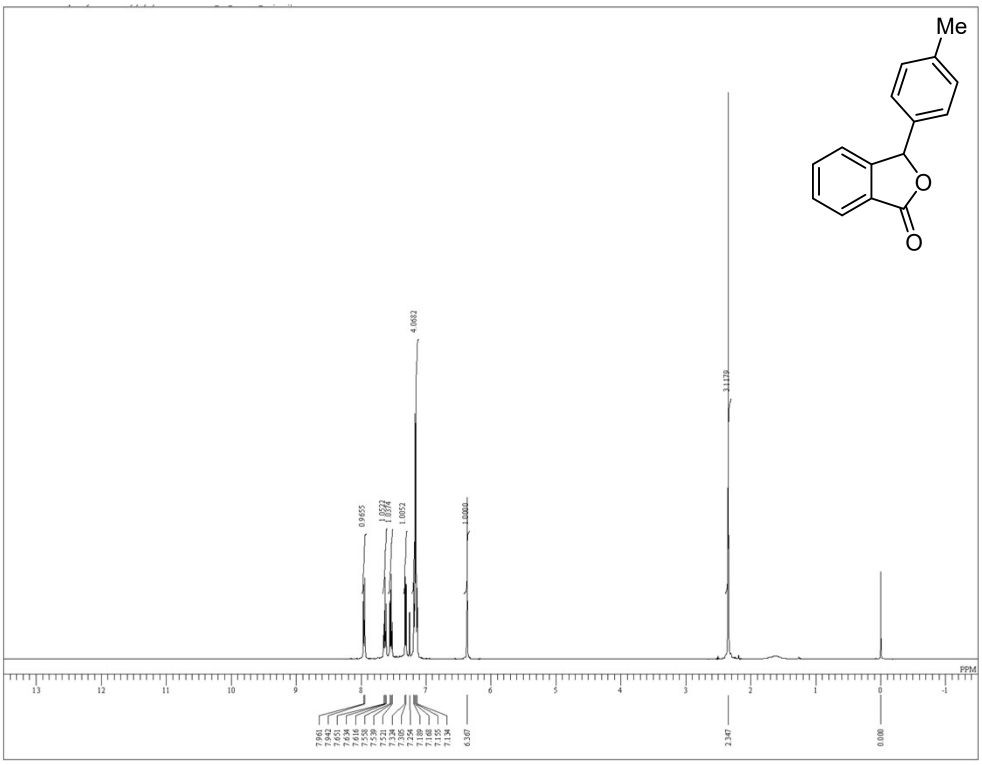

^{13}C{^{1}H} NMR (101 MHz, $CDCl_3$) of **2b**

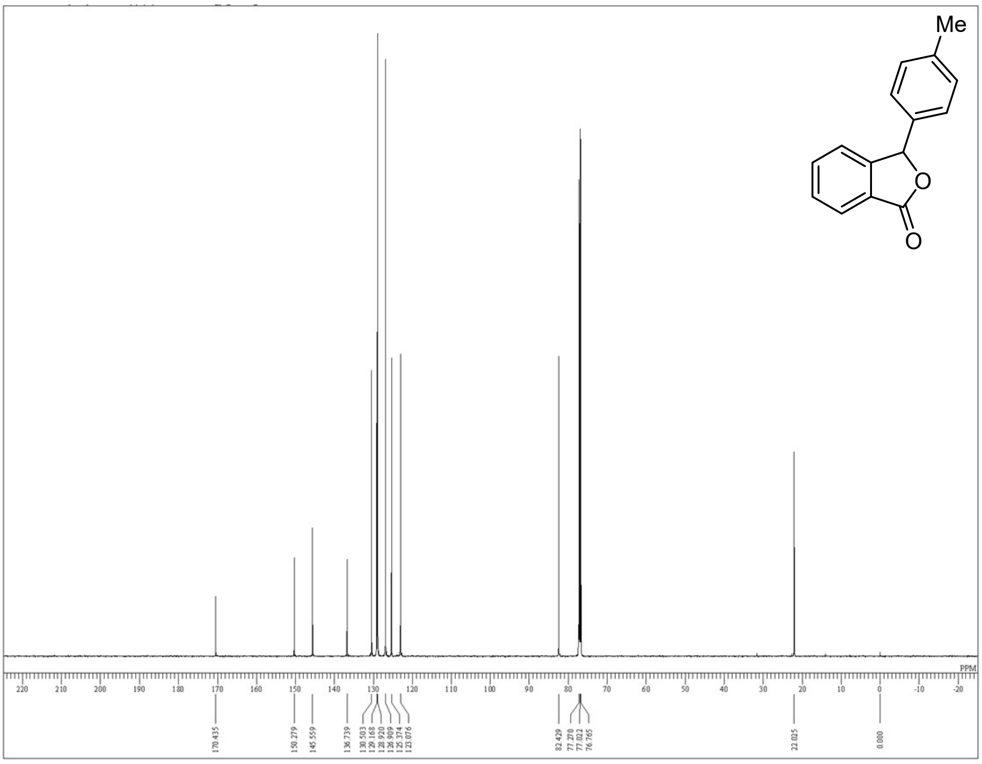

^{1}H NMR (400 MHz, $CDCl_3$) of **2c**

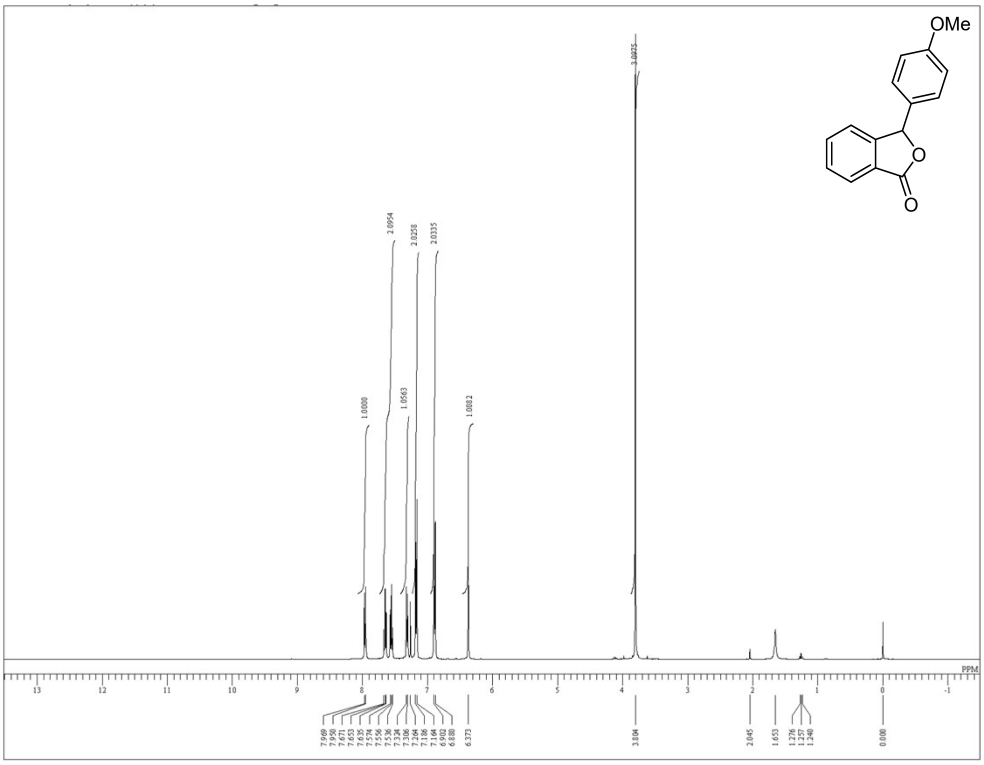

$^{13}C\{^{1}H\}$ NMR (101 MHz, $CDCl_3$) of **2c**

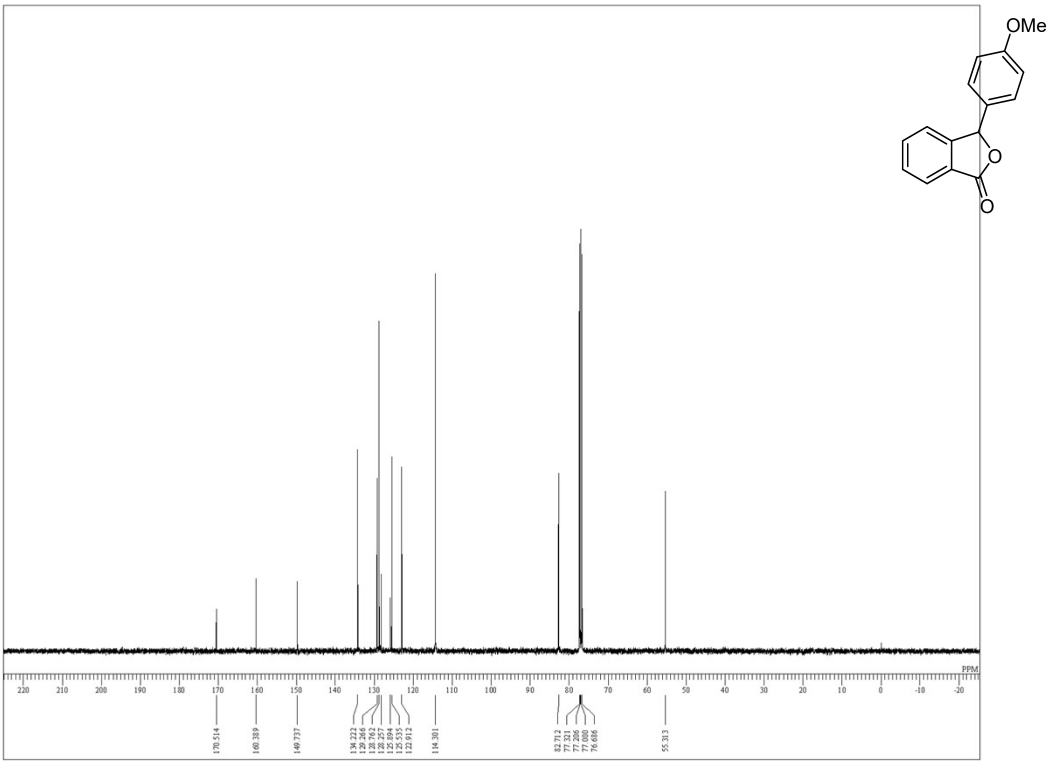

^{1}H NMR (400 MHz, $CDCl_3$) of **2d**

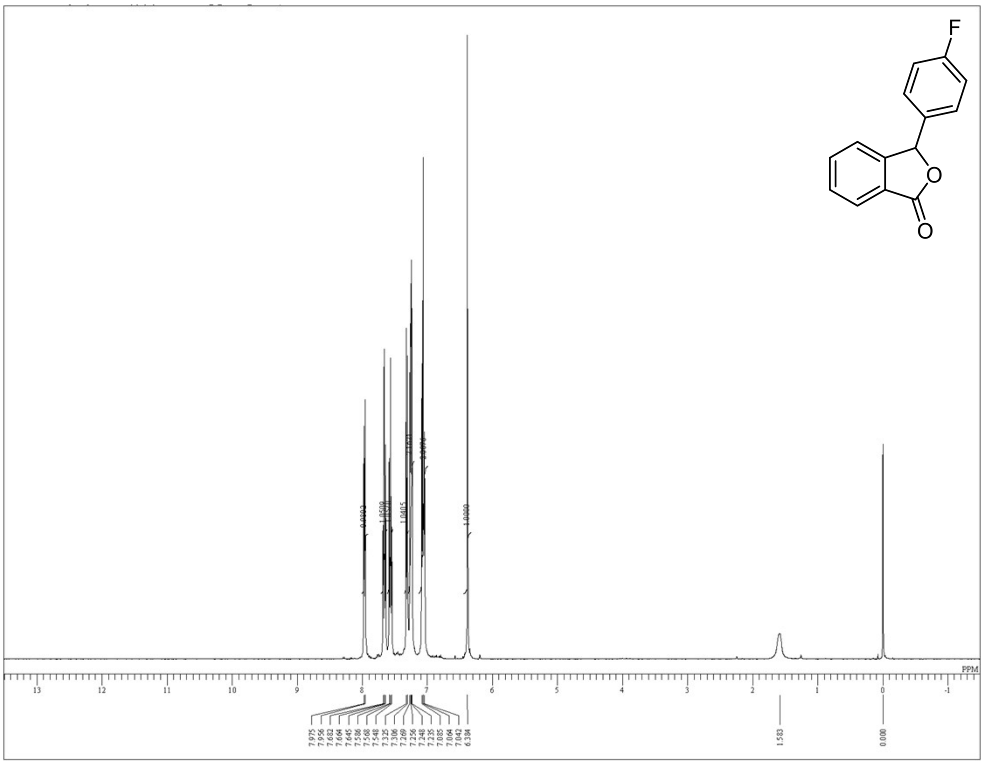

^{13}C{^{1}H} NMR (101 MHz, $CDCl_3$) of **2d**

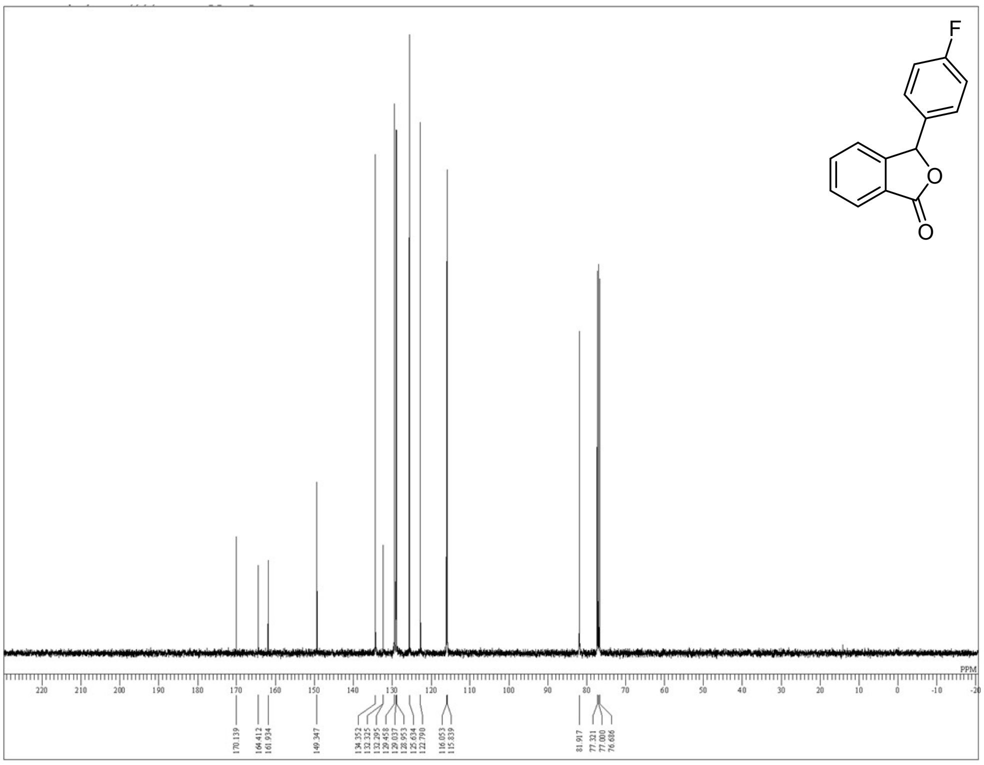

^{19}F NMR (376 MHz, $CDCl_3$) of **2d**

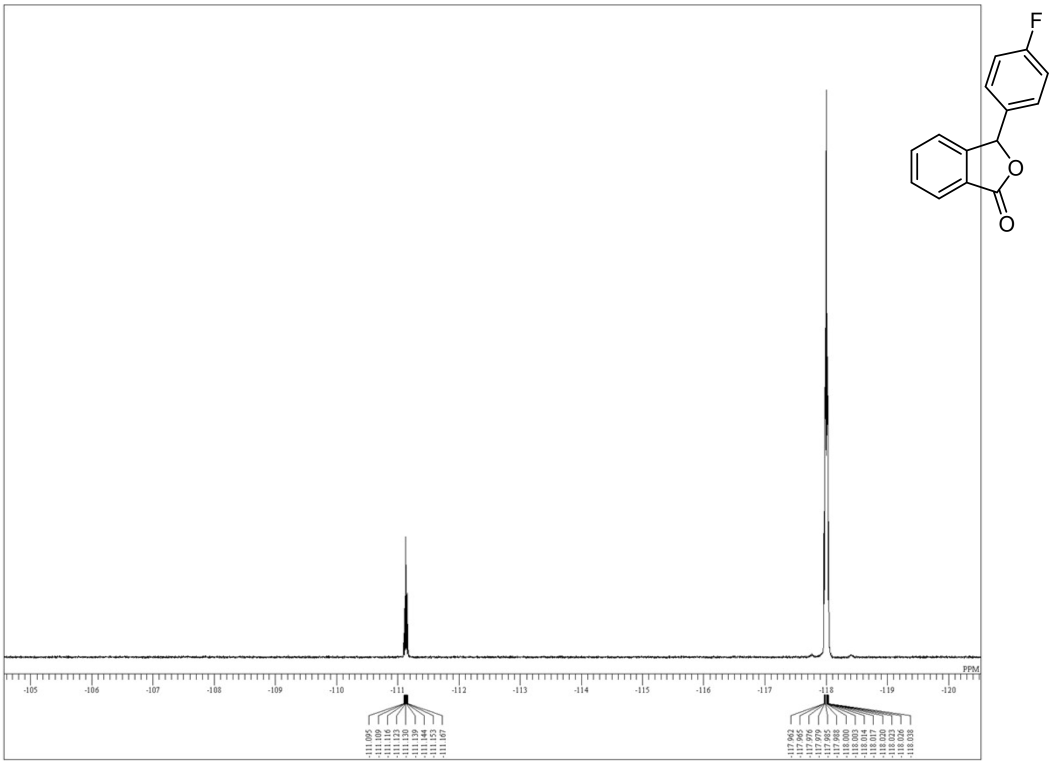

^{1}H NMR (400 MHz, $CDCl_3$) of **2e**

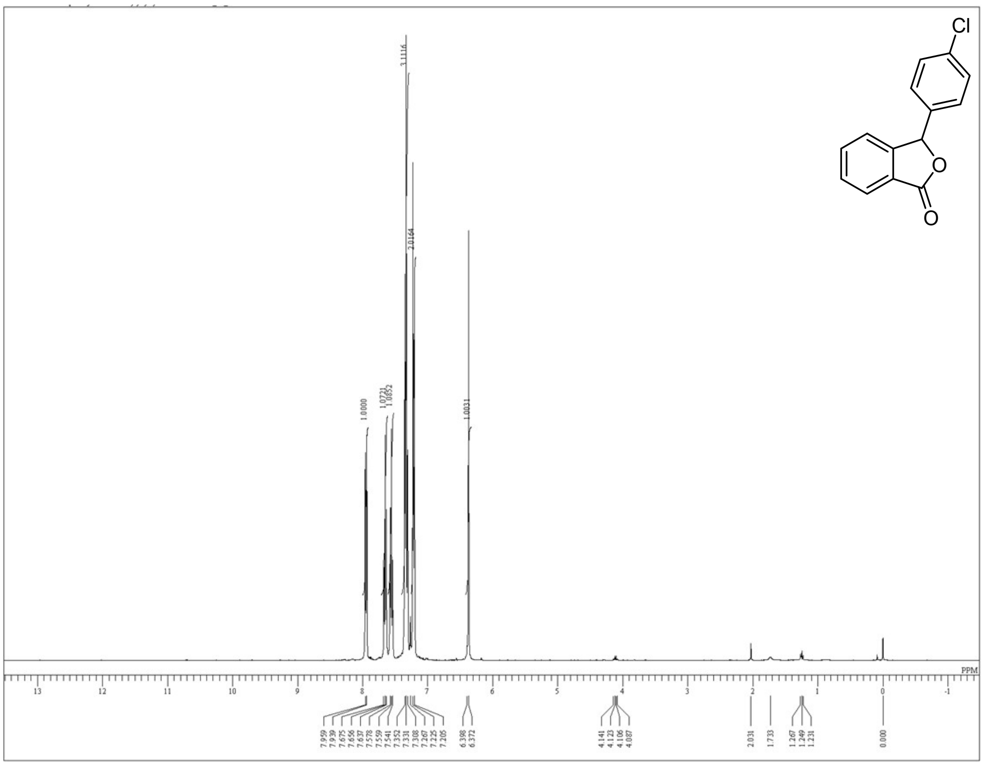

^{13}C{^{1}H} NMR (101 MHz, $CDCl_3$) of **2e**

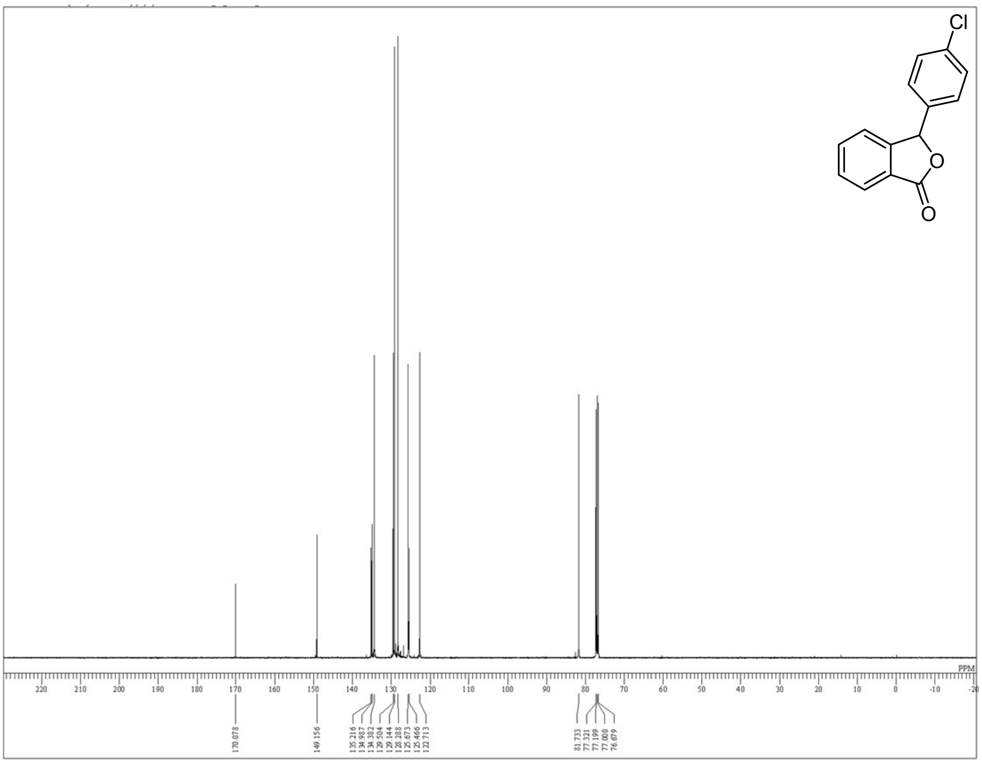

^{1}H NMR (400 MHz, $CDCl_3$) of **2f**

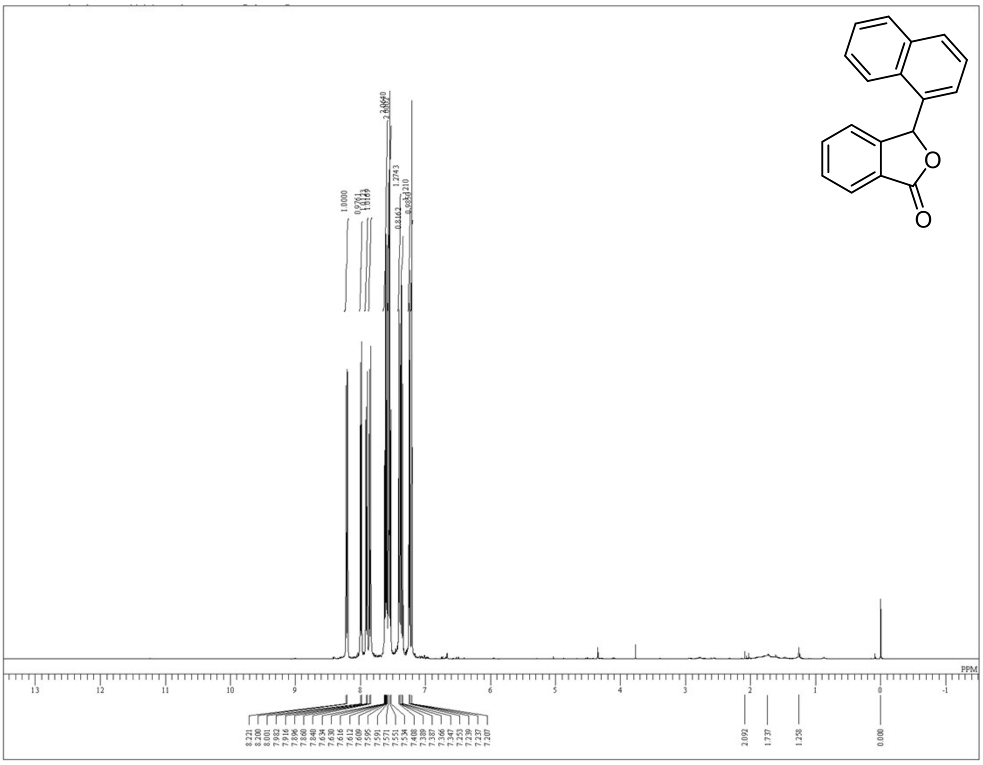

$^{13}C\{^{1}H\}$ NMR (101 MHz, $CDCl_3$) of **2f**

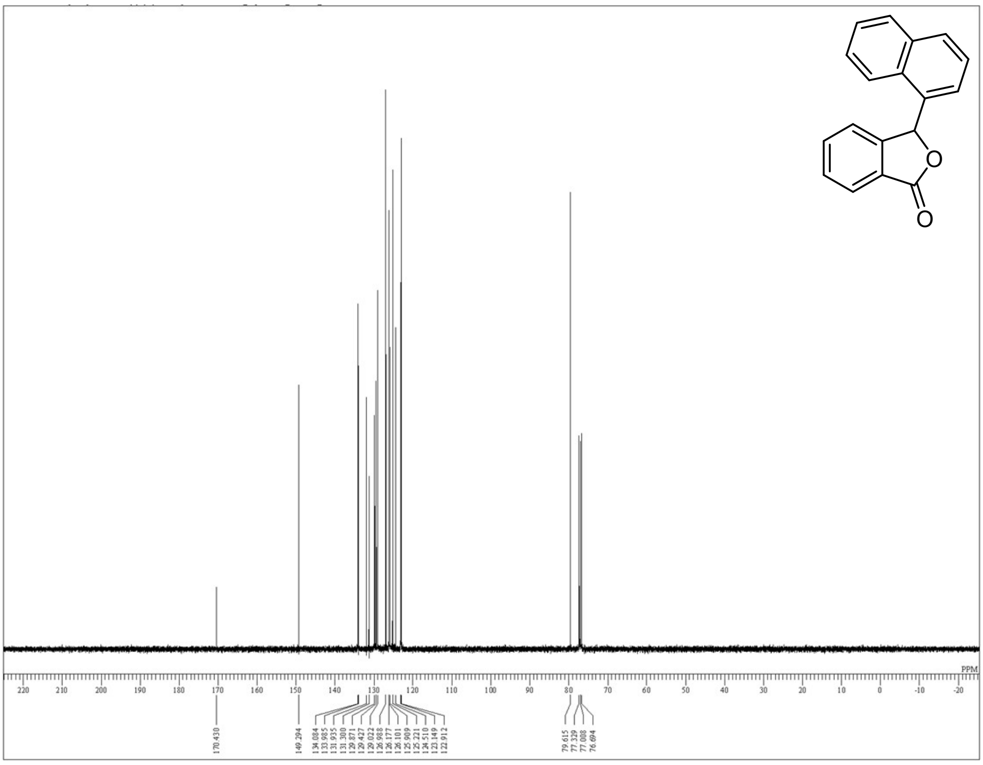

^{1}H NMR (400 MHz, $CDCl_3$) of **2g**

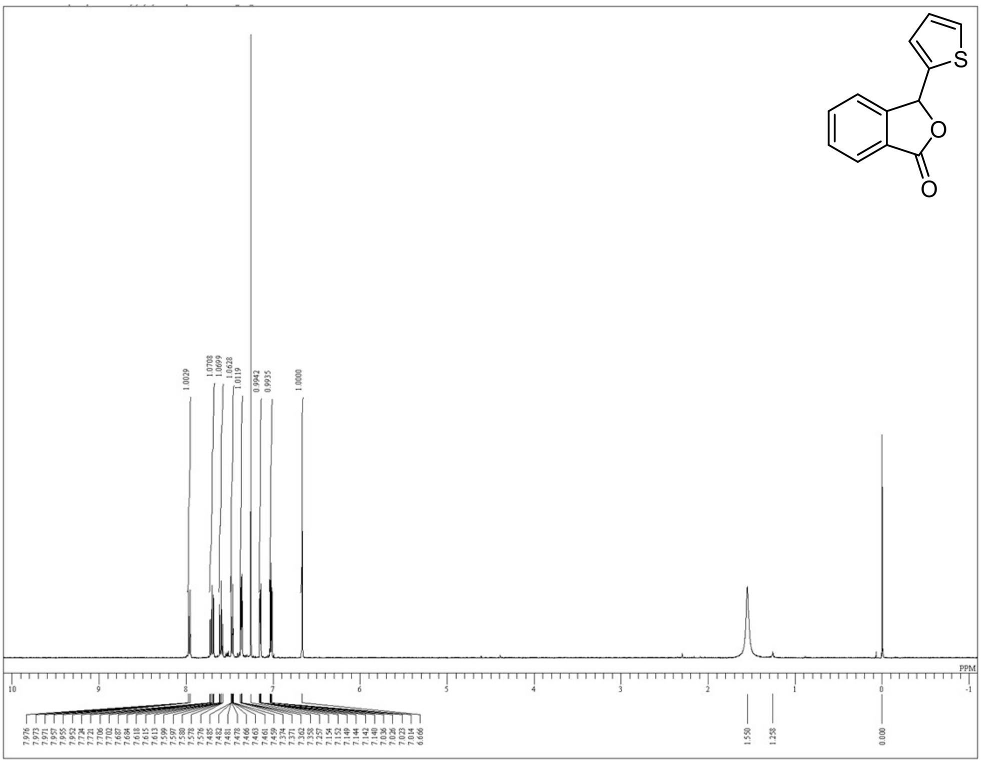

$^{13}C\{^1H\}$ NMR (126 MHz, $CDCl_3$) of **2g**

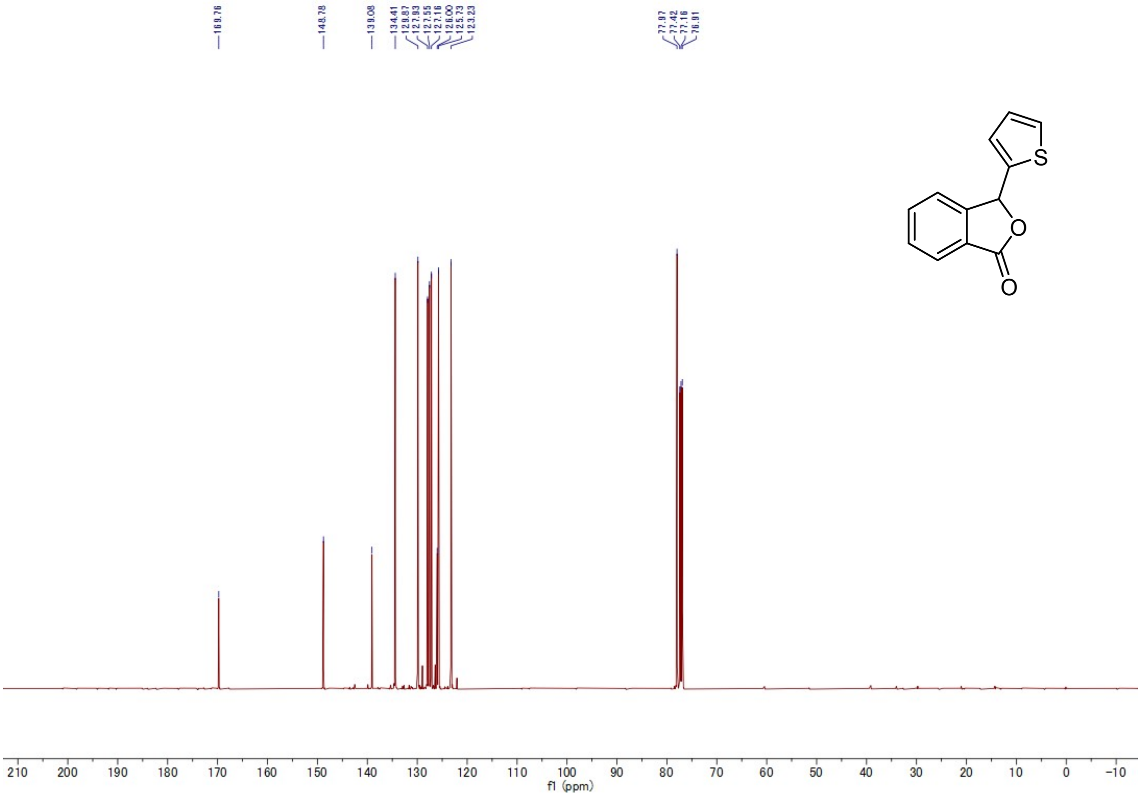

^{1}H NMR (400 MHz, $CDCl_3$) of **2i**

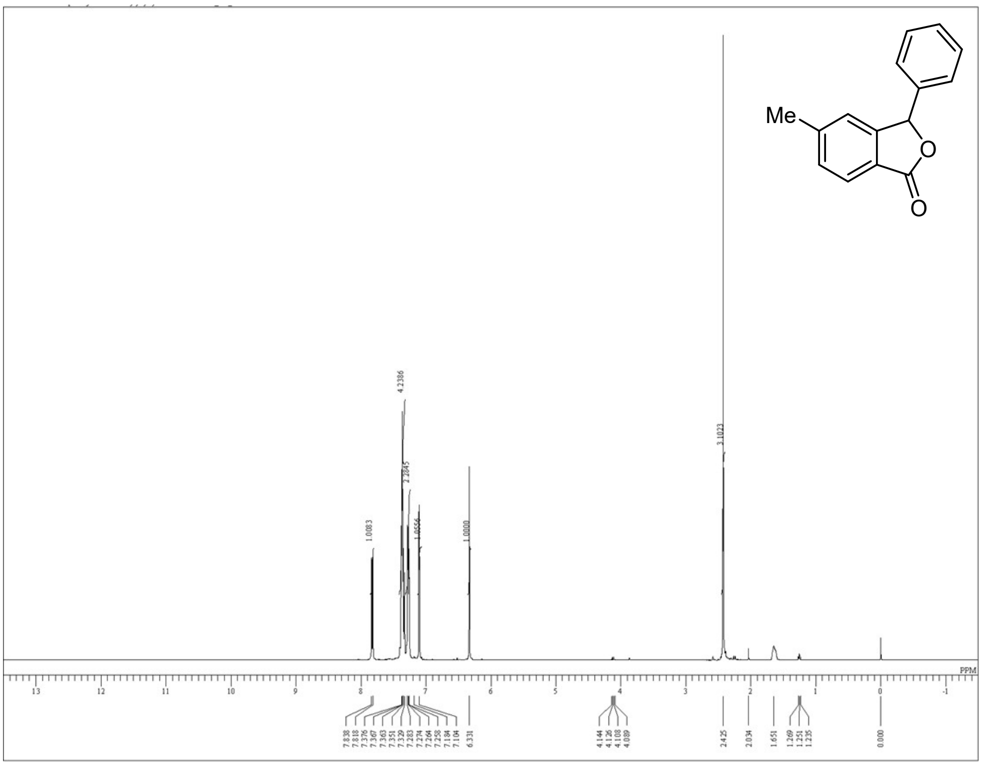

$^{13}C\{^{1}H\}$ NMR (101 MHz, $CDCl_3$) of **2i**

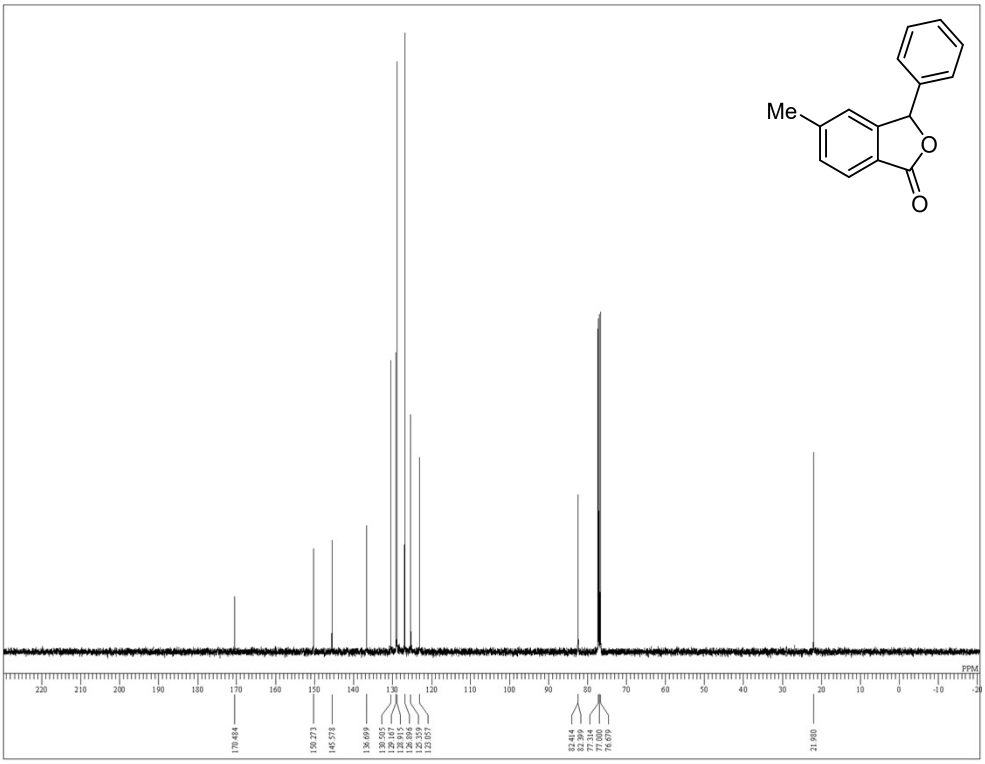

^{1}H NMR (400 MHz, $CDCl_3$) of **2j**

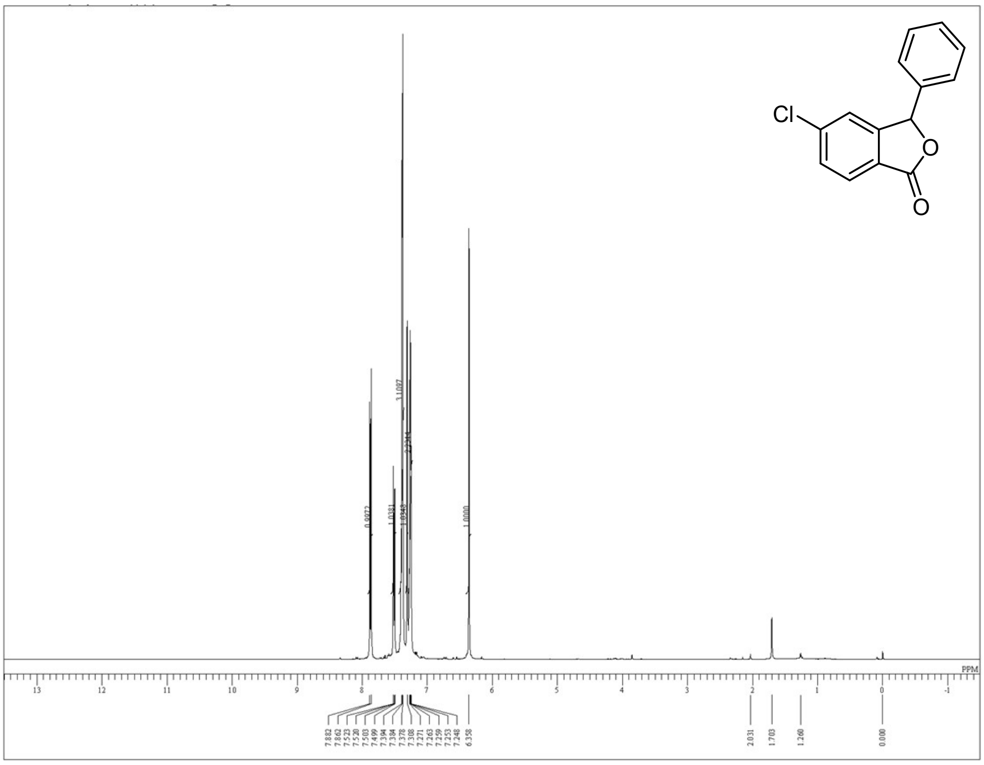

$^{13}C\{^{1}H\}$ NMR (101 MHz, $CDCl_3$) of **2j**

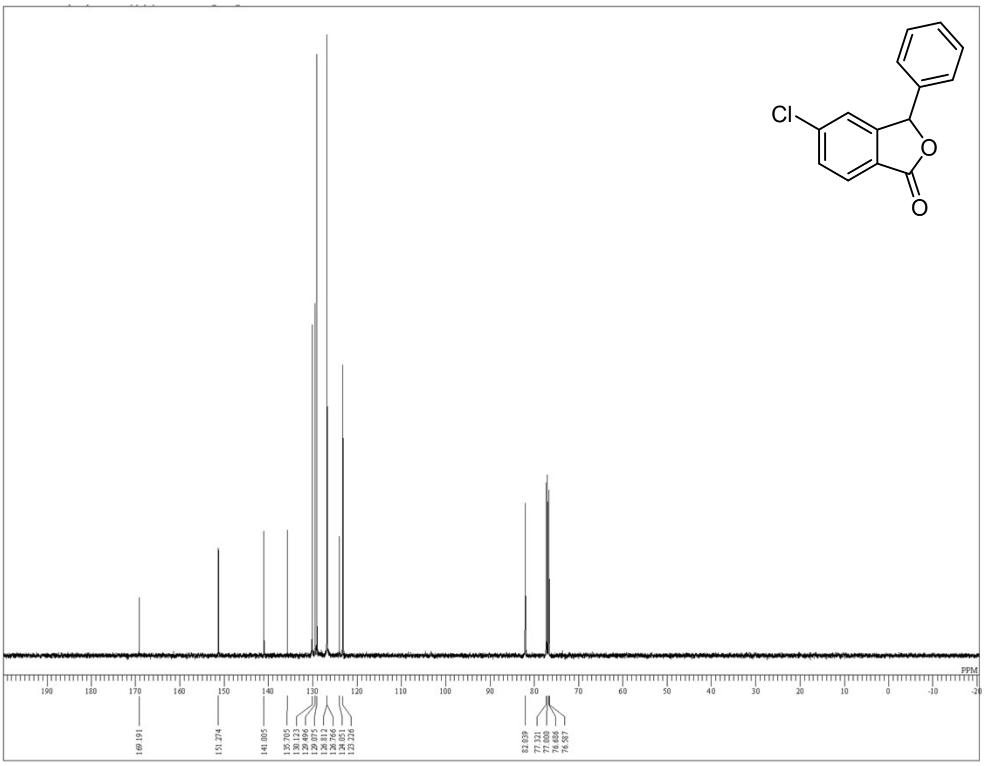

^{1}H NMR (400 MHz, $CDCl_3$) of **2k**

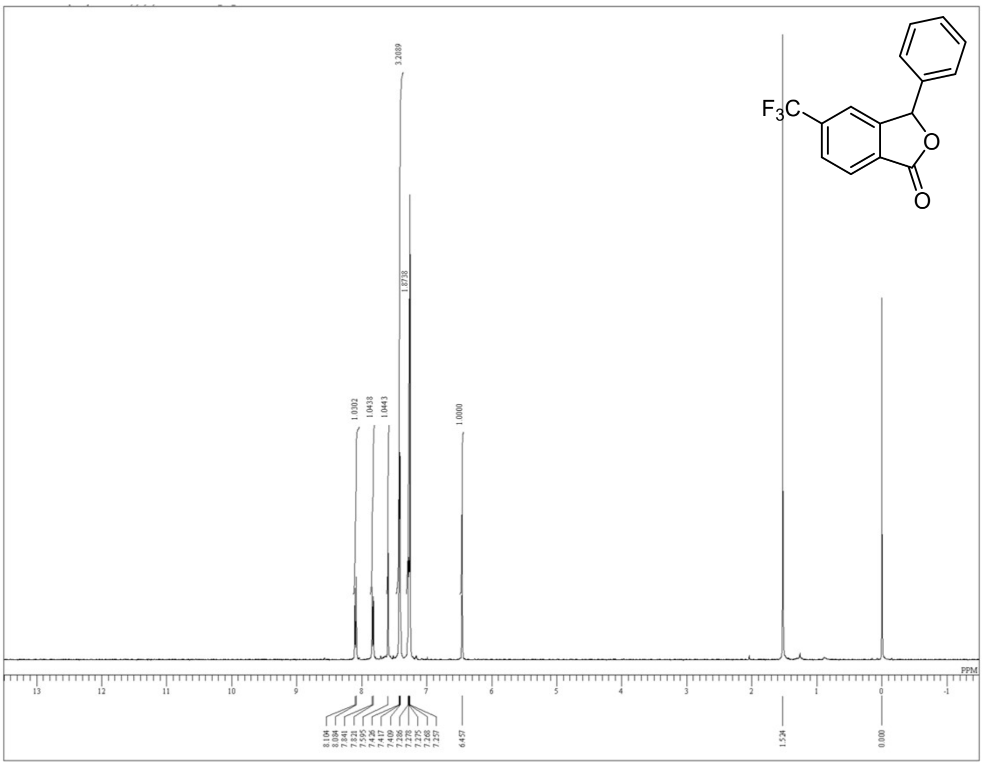

$^{13}C\{^{1}H\}$ NMR (101 MHz, $CDCl_3$) of **2k**

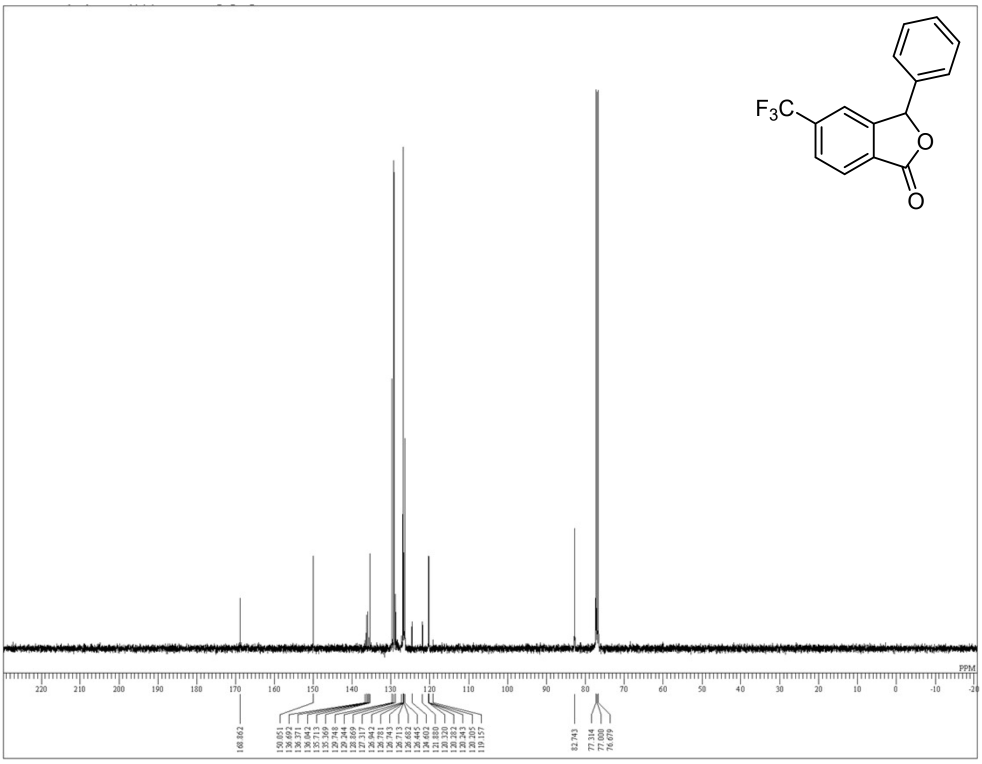

^{19}F NMR (376 MHz, $CDCl_3$) of **2k**

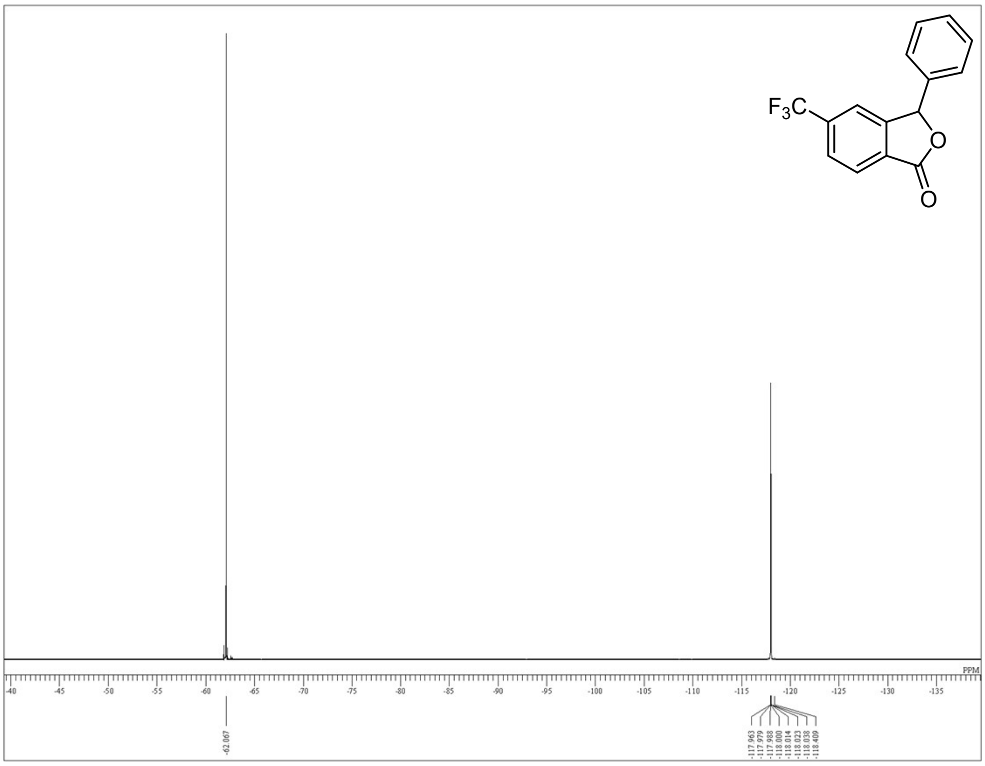

^{1}H NMR (400 MHz, $CDCl_3$) of **2l**

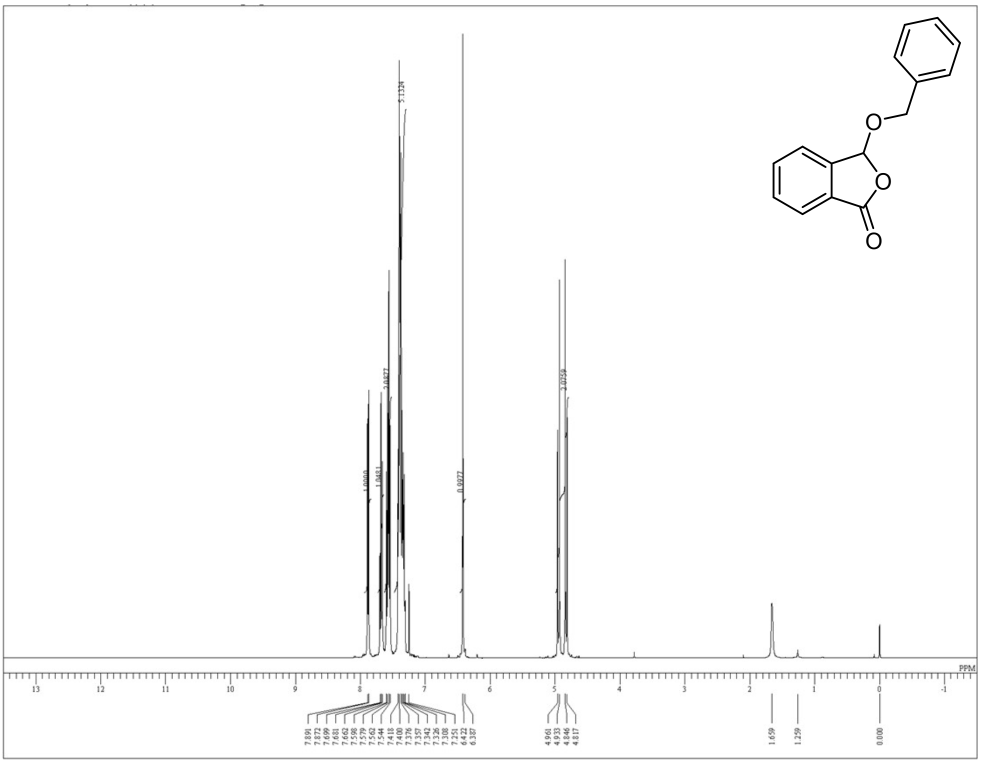

$^{13}C\{^{1}H\}$ NMR (101 MHz, $CDCl_3$) of **2l**

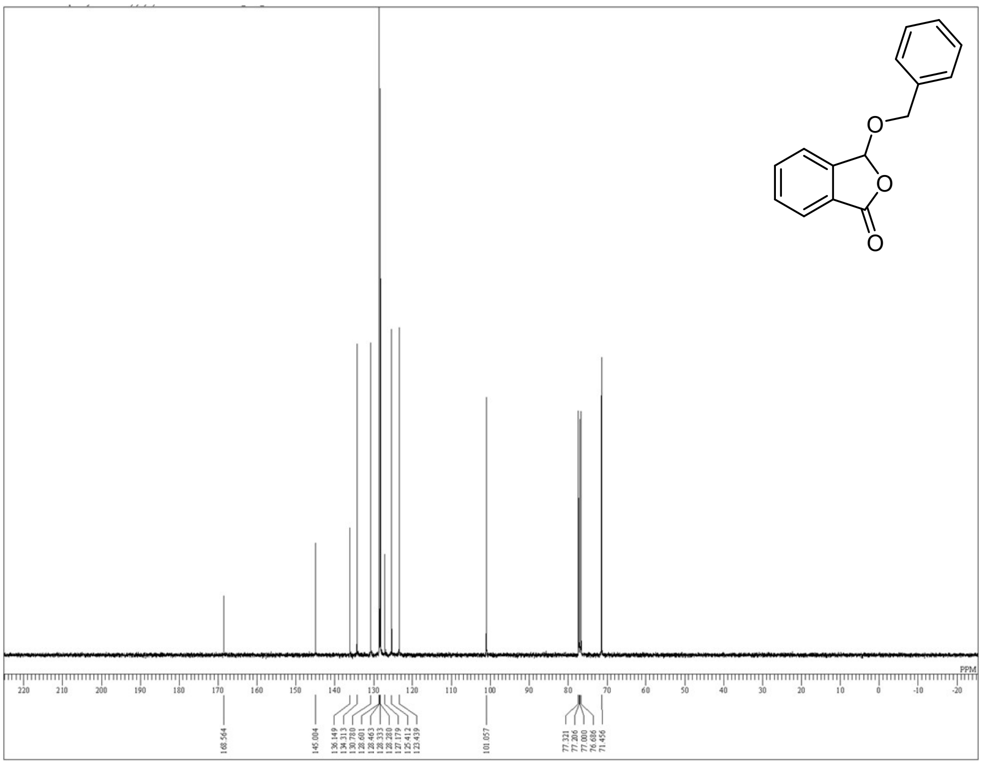

^{1}H NMR (400 MHz, $CDCl_3$) of **3**

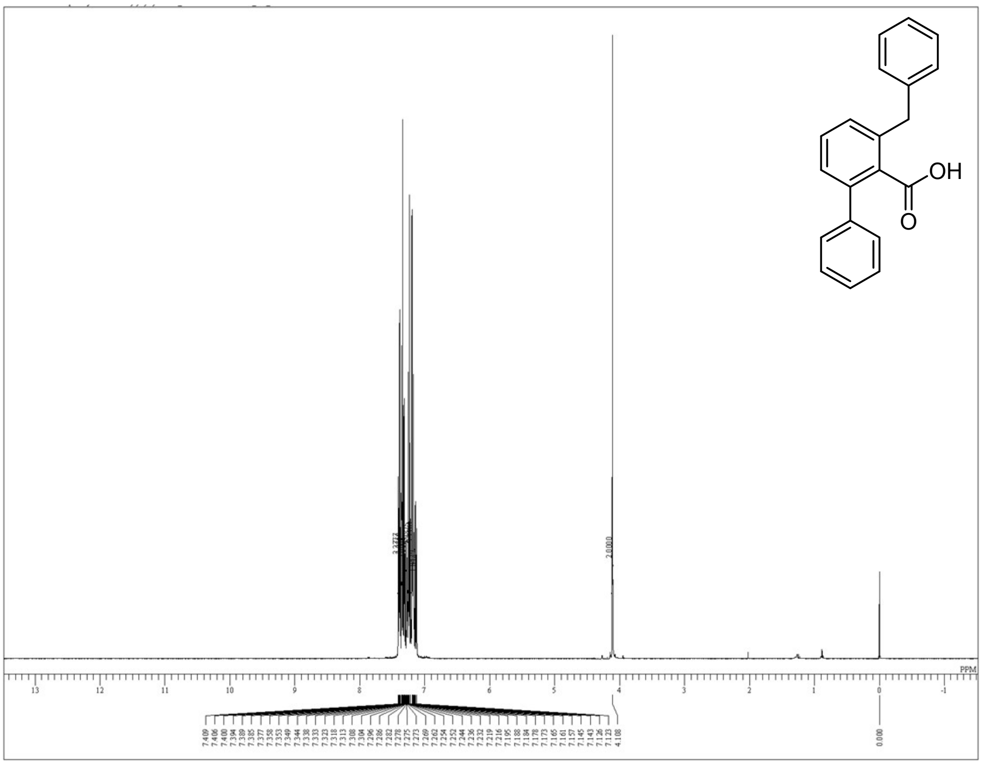

^{13}C{^{1}H} NMR (101 MHz, $CDCl_3$) of **3**

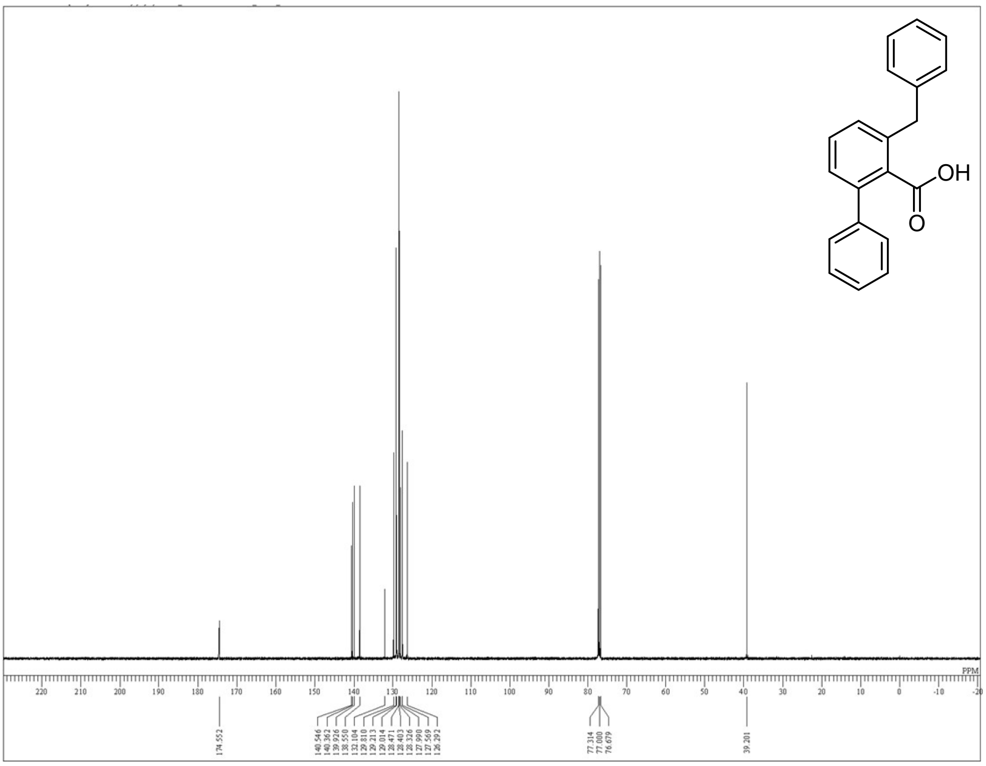

^{1}H NMR (400 MHz, $CDCl_3$) of **1h**

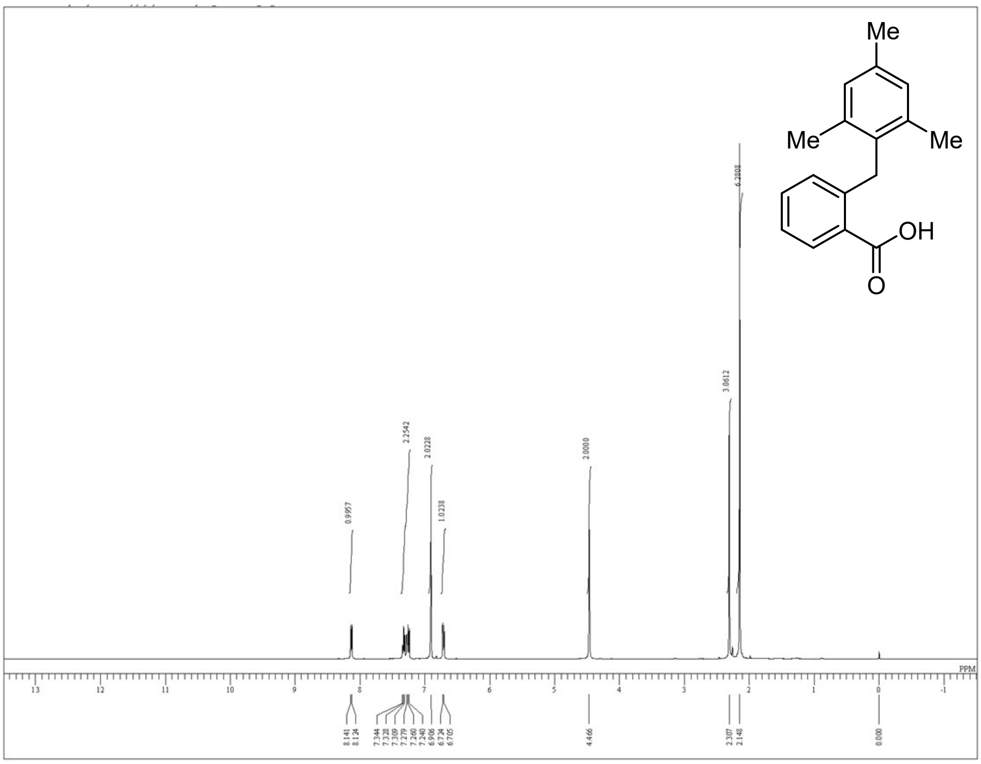

^{13}C{^{1}H} NMR (101 MHz, $CDCl_3$) of **3**

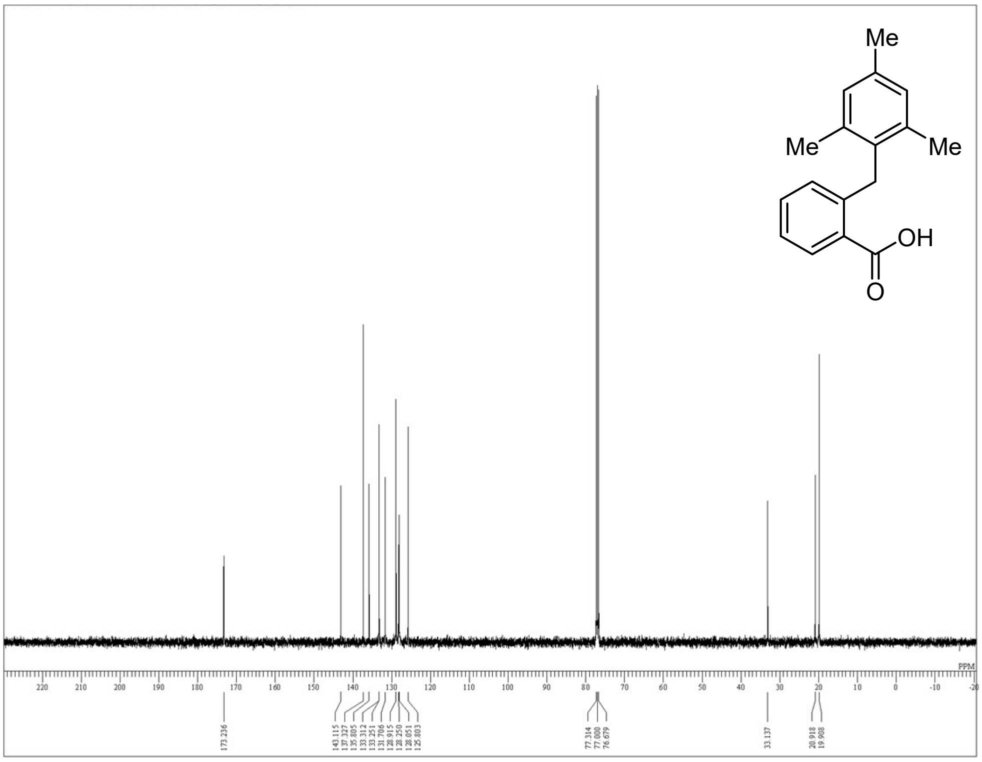

^{1}H NMR (400 MHz, $CDCl_3$) of **1i**

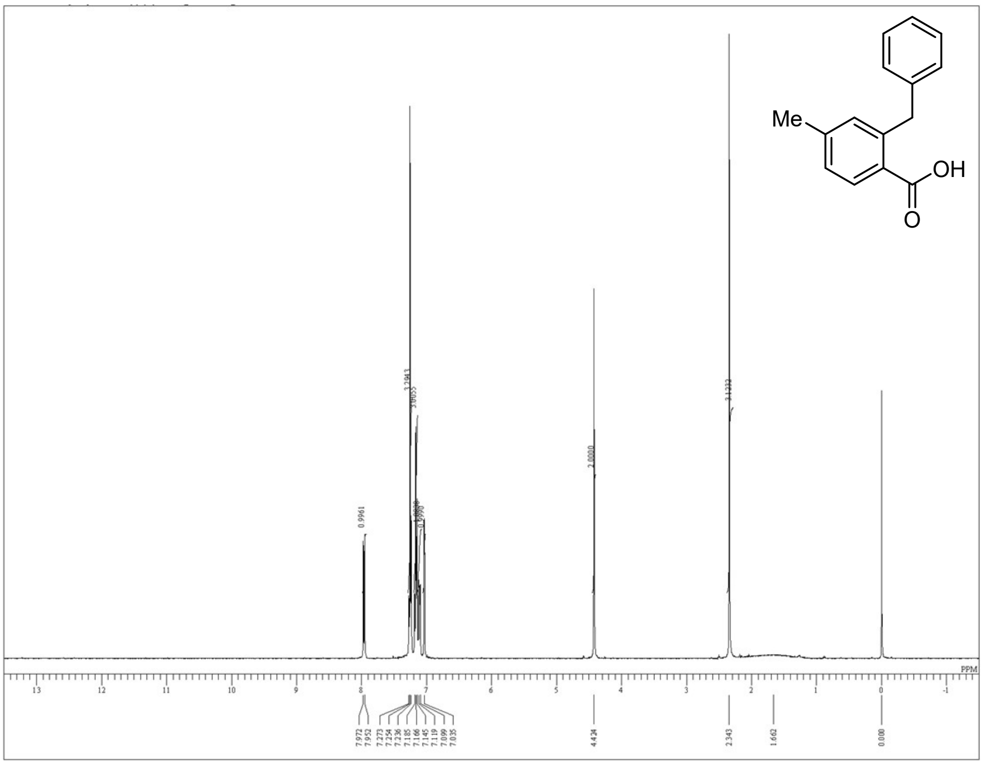

^{13}C{^{1}H} NMR (101 MHz, $CDCl_3$) of **1i**

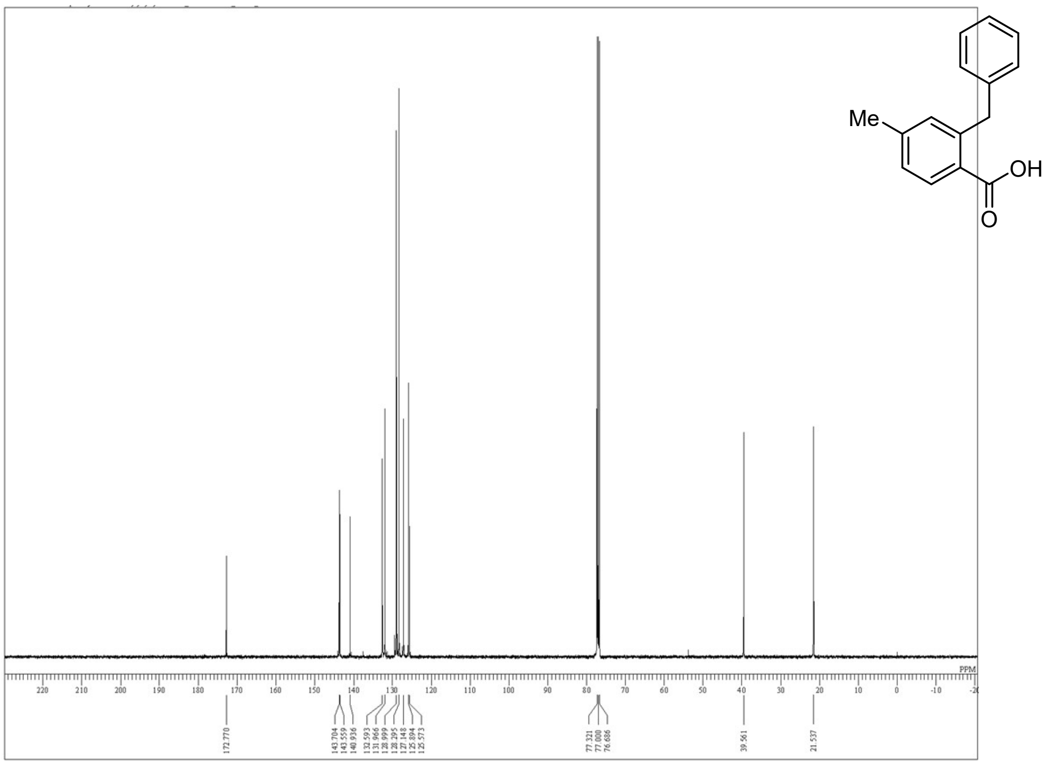

^{1}H NMR (400 MHz, $CDCl_3$) of **1j**

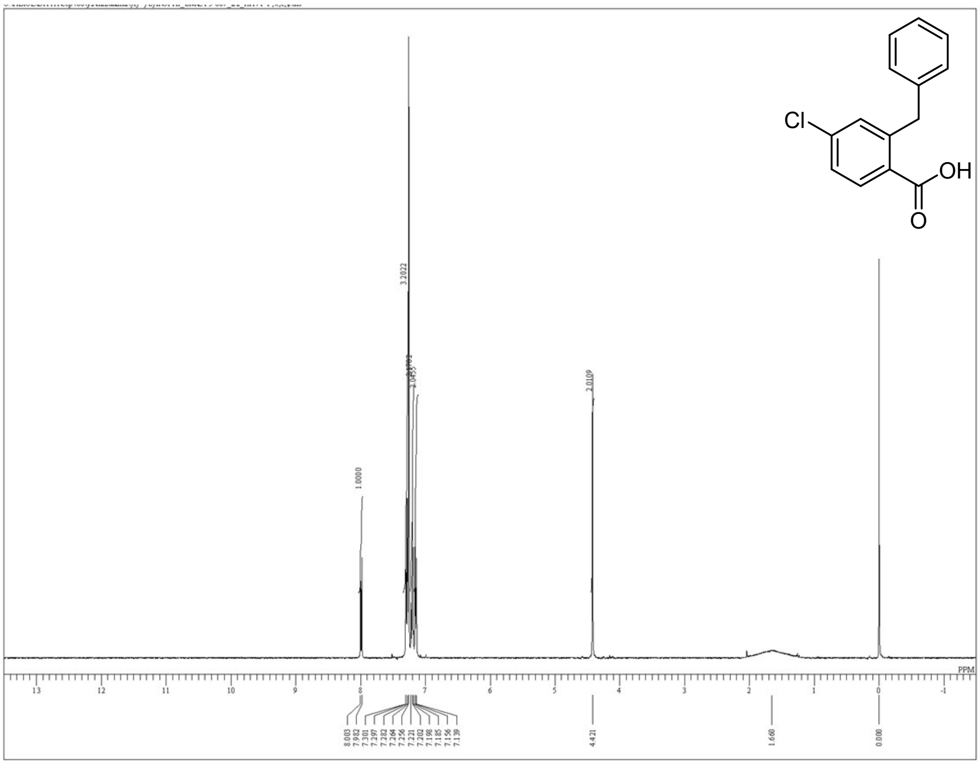

$^{13}C\{^{1}H\}$ NMR (101 MHz, $CDCl_3$) of **1j**

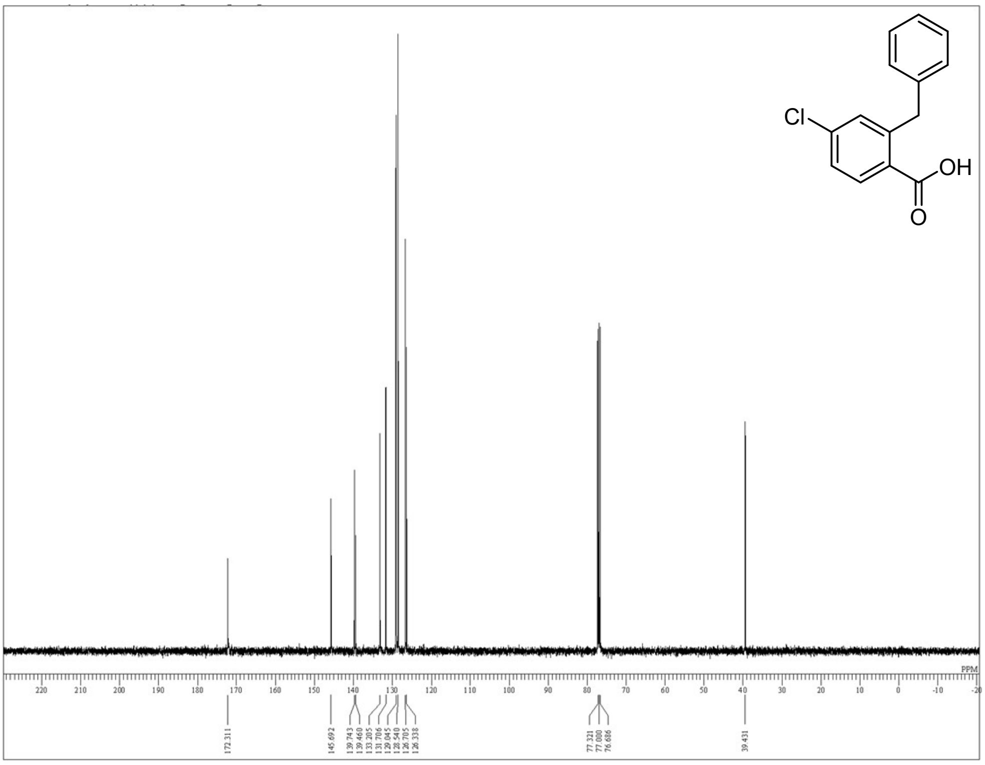

^{1}H NMR (400 MHz, $CDCl_3$) of **1k**

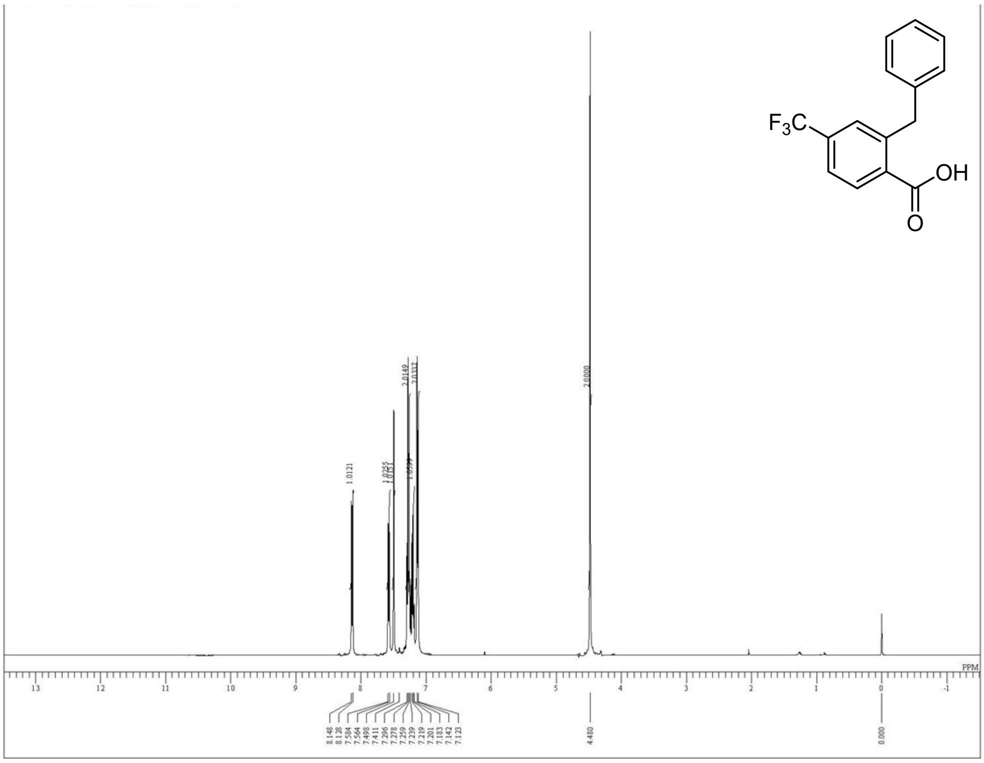

$^{13}C\{^{1}H\}$ NMR (101 MHz, $CDCl_3$) of **1k**

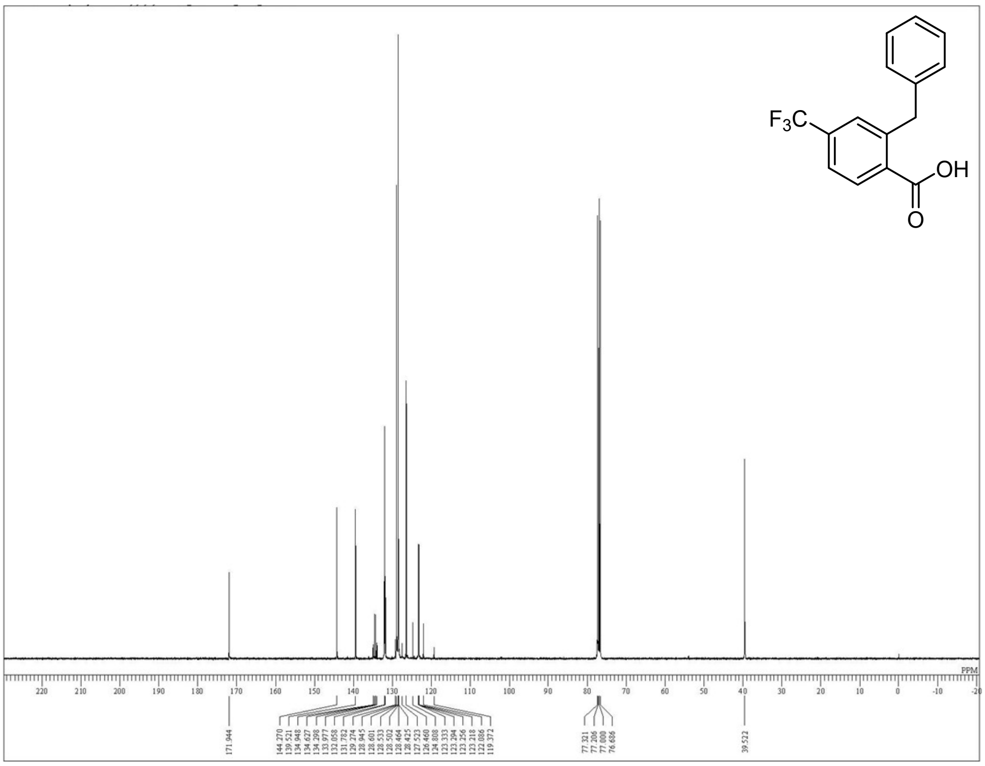

^{19}F NMR (376 MHz, $CDCl_3$) of **1k**

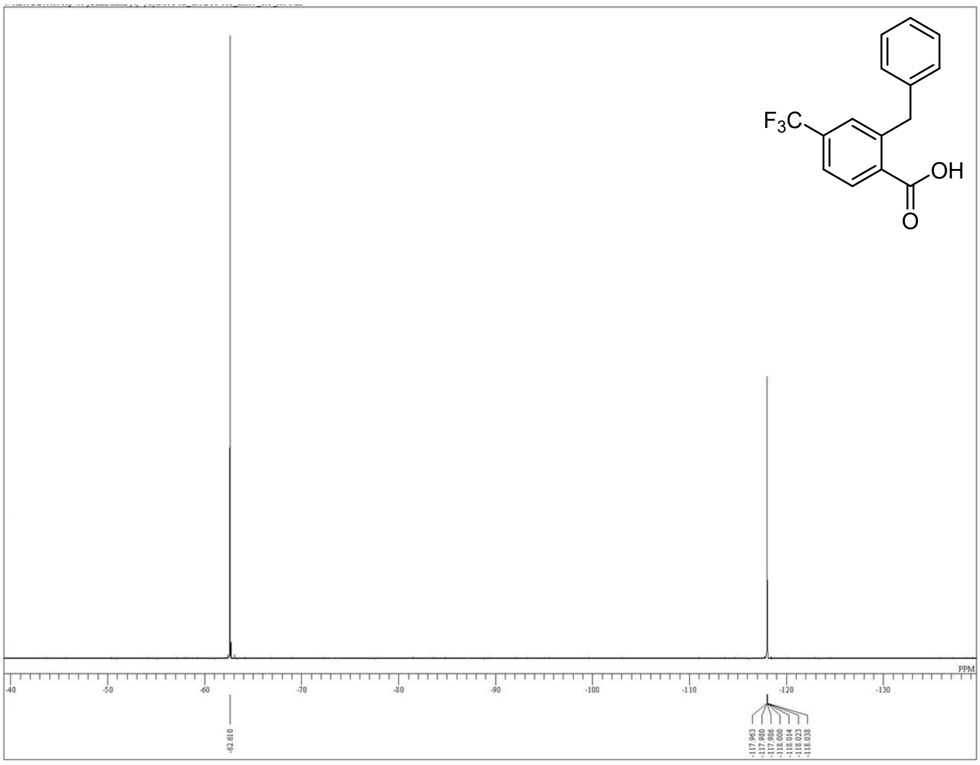

Supplement: RA-014-D3RA08176A-s001 [file RA-014-D3RA08176A-s001.pdf]
